# Design, Synthesis, and Antifungal Activity of Novel Aryl-1,2,3-Triazole-β-Carboline Hybrids

**DOI:** 10.3390/molecules23061344

**Published:** 2018-06-04

**Authors:** Xin-Yu Huo, Liang Guo, Xiao-Fei Chen, Yue-Ting Zhou, Jie Zhang, Xiao-Qiang Han, Bin Dai

**Affiliations:** 1 School of Chemistry and Chemical Engineering, Key Laboratory for Green Processing of Chemical Engineering of Xinjiang Bingtuan, Shihezi University, Shihezi 832003, China; shz_hxy1993@sina.com (X.-Y.H.); guoliang_xj@163.com (L.G.); mykoob@163.com (X.-F.C.); 2 Key Laboratory at Universities of Xinjiang Uygur Autonomous Region for Oasis Agricultural Pest Management and Plant Protection Resource Utilization, College of Agricultural, Shihezi University, Shihezi 832003, China; zhouyueting@stu.shzu.edu.cn

**Keywords:** β-carboline, 1,2,3-trizole, antifungal activity, structure–activity relationships

## Abstract

The copper catalytic azide and terminal alkyne cycloaddition reaction, namely “click chemistry”, gives a new and convenient way to create l,4-disubstitutd-l,2,3-triazoles. In this work, 2-pyrrolecarbaldiminato–Cu(II) complexes were established as efficient catalysts for the three-component 1,3-dipolar cycloaddition reaction of arylboronic acid and sodium azide (NaN_3_) with terminal alkynes in ethanol at room temperature to 50 °C, 1,4-disubstituted 1,2,3-triazoles were synthesized. Following the optimized protocol, two series of new aryl-1,2,3-triazole-β-carboline hybrids have been designed and synthesized, and the chemical structures were characterized by ^1^H NMR, ^13^C NMR, and high-resolution mass spectrometry (HRMS). All of the target compounds were evaluated in vitro for their antifungal activity against *Rhizoctorzia solani*, *Fusarium oxysporum*, *Botrytis cinerea* Pers., sunflower sclerotinia rot, and rape sclerotinia rot by mycelia growth inhibition assay at 50 μg/mL. The antifungal evaluation of the novel hybrids showed that, among the tested compounds, **5a**, **5b**, **5c**, and **9b** showed good antifungal activity against sunflower sclerotinia rot. Specifically, compound **9b** also exhibited high broad-spectrum fungicidal against all the tested fungi with inhibition rates of 58.3%, 18.52%, 63.07%, 84.47%, and 81.23%. However, for *F. oxysporum*, all the target compounds showed no in vitro antifungal activities with an inhibition rate lower than 20%. These results provide an encouraging framework that could lead to the development of potent novel antifungal agents.

## 1. Introduction

Plant pathogenic microorganisms could infect crops and cause local or whole plant disease, which leads to significant economic losses [1]. In recent years, the potential impact of synthetic pesticides on the environment and human health has been of great concern, which highlights the need for environmentally-friendly pesticides to protect crops from insect infestation [2]. Therefore, plant-derived extracts and their bioactive natural compounds have been considered bio-rational alternatives [3]. Additionally, further modification and structural optimization of novel insecticides leading from the plant origin have recently been important methods for the research and development of new pesticides [4]. Harmine, harman, and harmol, belonging to the β-carboline alkaloid class, are present in medicinal plants, such as *Peganum harmala* L. [5]. The reported biological applications of β-carboline alkaloids include sedative and anxiolytic [6], antitumor [7,8], antimalarial [8], antiparasitic [9], anti-HIV [10] agents, and other pharmacological activities. As for pest management, the extracts of *Peganum harmala* L. plant species containing a mixture of harmine, harmaline, and norharman, as well as their derivatives, had been proven to have excellent insecticidal, fungicidal, and plant growth regulatory properties [11,12,13,14,15,16,17]. In our previous work [18], we found that 9-fluorosubstituted-harmine displayed higher fungicidal activities against *Rhizoctonia solani*, *Rape sclerotinia* rot, and *Alternaria kikuchiana* Tanaka.

l,2,3-Triazole and its derivatives as an important class of nitrogen-containing aromatic heterocyclic compounds have attracted a great deal of interest due to their diverse biological activities, such as anticancer [19,20] and antifungal [20] activities, and other properties [21,22]. Meanwhile, the 1,2,3-triazole moiety is stable with regard to metabolic degradation, and capable of hydrogen bonding, which could be favorable in binding of biomolecular targets and increasing solubility [23]. Moreover, 1,2,3-triazoles can be attractive as linker units, which could connect two pharmacophores to give an innovative bifunctional drug, and have become increasingly useful and important in constructing bioactive molecules [24,25].

**Figure 1:**
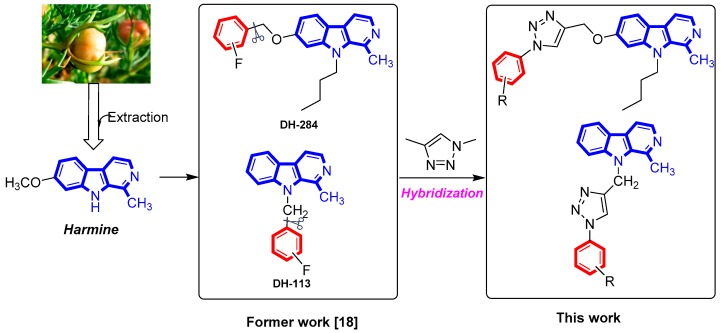
Designed strategy of aryl-1,2,3-triazole-β-carboline hybrids.

Accordingly, in an attempt to improve activity of β-carboline derivatives, in this paper, we synthesized two series of novel aryl-1,2,3-triazole-β-carboline hybrids (see Figure 1). Their antifungal activities were evaluated in vitro.

## 2. Results and Discussion

### 2.1. Chemistry

**Scheme 1:**
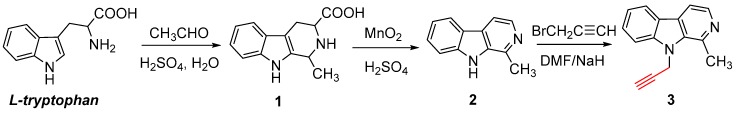
Synthesis of the key intermediate **3**.

The synthesis of the desired key intermediate 1-methyl-9-(prop-2-yn-1-yl)-β-carboline (**3**) was performed in three steps starting from L-tryptophan, which was outlined in Scheme 1. The synthetic step involved the Pictet–Spengler condensation [6], and was followed by oxidation and decarboxylation to afford the intermediate 1-methyl-β-carboline (**2**). In the next step, the *N*^9^-alkylated of compound **2** was prepared by the action of sodium hydride (NaH) in anhydrous *N*,*N*-dimethylformamide (DMF) followed by addition of propargyl bromide to afford compound **3**, which incorporates an alkynyl group required for click chemistry.

A number of synthetic methodologies [19,26,27] are available in the literature for the synthesis of 1,2,3-triazole. In our previous investigation [28,29], we have found that 2-pyrrolecarbaldiminato-Cu(II) complexes are efficient catalysts, which affords the 1-benzyl-1,2,3-triazoles in good yields. In order to improve the selectivity of the reaction, we have studied the reaction conditions by screening various catalysts. Initially, the cycloaddition reaction between phenylboronic acid, NaN_3_, and 1-methyl-9-propargyl-β-carboline (**3**) was selected as a model reaction to investigate the catalytic activity of four different 2-pyrrolecarbaldiminato–Cu(II) complexes, and the results are summarized in Table 1. It was found that the azidonation reaction of phenylboronic acid with NaN_3_ proceeded smoothly within 8 h in the presence of the four Cu(II) complexes with 1 mol % loading. Subsequently, we added intermediate **3** to the reaction mixture, and the solution was heated at 50 °C for 2 h. The click cyclization reaction was completed to give the 1,4-disubstituted 1,2,3-triazoles in the yields of 69% to 84%, and Cu(II)-complex L**_1_** was found to be the best (Entries 1–4). The control experiment indicated that the reaction could not occur without the Cu(II)-complex (Entry 5). When the amount of the Cu(II)-complex, L**_1_**, was reduced from 1 mol % to 0.5 mol %, it resulted in a lower yield (Entry 6). Therefore, the optimal conditions for aryl-1,2,3-triazole-β-carboline hybrid synthesis involves the use of 1 mol % Cu(II)-complex L**_1_** as the catalyst. and ethanol as the solvent.

**Scheme 2:**
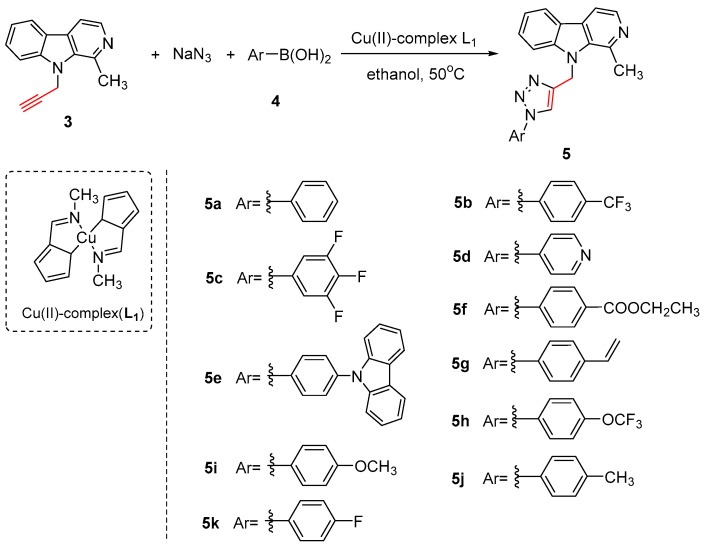
Synthesis of the 9-(1,2,3-triazolyl)-β-carboline hybrids **5a**–**k**.

**Scheme 3:**
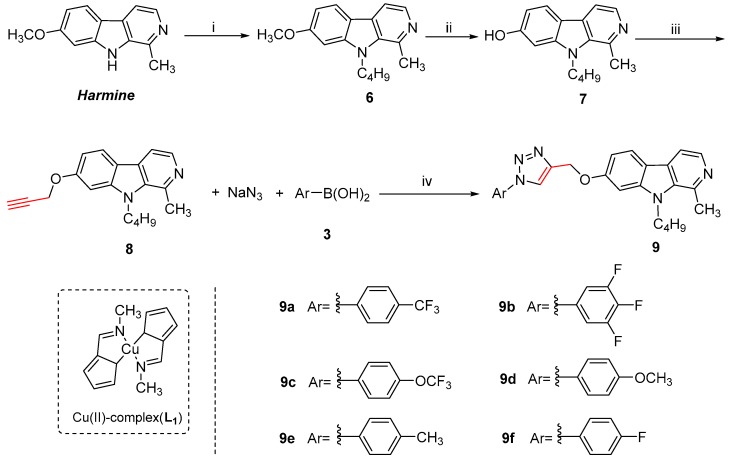
Synthesis of the 7-(1,2,3-triazolyl)-β-carboline hybrids **9a**–**f**. **Reagents and conditions:** (i) DMF, NaH, *n*-iodobutane, stirred at RT; (ii) HBr, HOAc, reflux (iii) DMF, NaH, 3-bromopropyne, stirred at RT; (iv) Cu(II)-complex L**_1_**, ethanol, 50 °C.

The generality of the optimized reaction condition was studied with a wide range of substrates, using various substituted phenylboronic acid bearing electron-withdrawing and electron-donating substituents, NaN_3_, and 1 mol % Cu(II)-complex L**_1_** with 1-methyl-9-propargyl-β-carboline **3** to afford 9-(1,2,3-triazolyl)-β-carboline hybrids **5a**–**k**, which are shown in Scheme 2. The synthetic routes of novel 7-(1,2,3-triazolyl)-β-carboline hybrids **9a**–**f** are outlined in Scheme 3. The *N*^9^-alkylated harmine derivative **6** was prepared according to the synthetic protocol described by our group [30]. The preparation of compound **7** followed a common synthetic scheme and was characterized by demethylation of compound **6** using hydrobromic acid and acetic acid as the reaction solvent. Compound **8**, bearing alkoxy in postion-7 of β-carboline core, was synthesized from compound **7** by the action of NaH in dry DMF followed by addition of propargyl bromide in 81% yield. Lastly, the synthesis of compounds **9a**–**f** was carried out following the general procedure for the synthesis of compounds **5a**–**k**. All structures of the final products were determined by ^1^H NMR, ^13^C NMR (see Supplementary Materials), and HRMS.

### 2.2. Fungicidal Activities

From the synthetic route mentioned above, we obtained two series of novel aryl-1,2,3-triazole-β-carboline hybrids **5a**–**k**, **9a**–**f**. These compounds were evaluated in a series of fungicidal tests in vitro against a range of phytopathogenic species including *R. solani*, *Fusarium oxysporum*, *Botrytis cinerea* Pers., sunflower sclerotinia rot, and rape sclerotinia rot. The activity results obtained as an inhibition rate are summarized in Table 2.

Generally, at 50 μg/mL, the target compounds exhibited different levels of antifungal activity against these five tested fungi. Compared with that of the commercial fungicide carbendazim and azoxystrobin, these compounds have exhibited a significant inhibitory effect against sunflower sclerotinia rot (SCR) in which compounds **5a** (Ar = phenyl), **5b** (Ar = 4-trifluoromethylphenyl), **5c** (Ar = 3,4,5-trifluorophenyl), and **9b** (Ar = 3,4,5-trifluorophenyl) had inhibitory rates of 85.04%, 86.93%, 85.98%, and 84.47%, respectively, which displayed comparable antifungal activity than that of the positive control, with an inhibition rate of 89.77% and 88.07%. In addition, compounds **5d**–**g**, **5i**–**k**, **9c**–**d**, and **9f** displayed moderate activity, with an inhibition rate ranging from 50% to 80%. For *F. oxysporum*, all the target compounds showed inactive in vitro antifungal activities with an inhibition rate lower than 20%. Similarly, for *R. solani*, the compounds showed weak antifungal activities with an inhibition rate ranging from 20% to 50%, except for **9b**, which exhibited moderate activity with an inhibition rate of 58.30%. However, it was not as clear as the one drawn from the RSR data. Some of the compounds exhibited significant activities in vitro toward RSR in which the compound **9b** had control efficacy rates of 81.23% and most of them showed weak to moderate activity. Of all aryl-1,2,3-triazole-β-carboline hybrids, compound **9b** displayed as broad a fungicidal spectrum as azoxystrobin and carbendazim against these phytopathogens.

## 3. Materials and Methods

### 3.1. General Information

All the reactions were monitored by TLC on silica gel F254 plates (Qingdao Haiyang Inc., Qingdao, China) for detection of the spot. Column chromatography was performed with silica gel (200–300 mesh). NMR spectra were recorded at room temperature on a Bruker Avance III HD 400 instrument at 400 MHz for ^1^H NMR and 100 MHz for ^13^C NMR (Bruker Company, Bremen, Gemany). CDCl_3_, DMSO-*d*_6_, Methanol-*d*_4_ or Pyridine-*d*_5_ was used as the solvent and TMS as the internal standard. High-resolution mass spectrometry (HRMS) were measured on Bruker ultrafleXtreme MALDI-TOF/TOF-MS and HCCA (alpha-cyano-4-hydroxycinnamic acid) is used as matrix.

All solvents were purified and dried using standard methods prior to use. The following intermediates, 1-methyl-β-carboline **2** [31], 7-methoxy-9-*n*-butyl-1-methyl-β-carboline **6 [30]** and 9-*n*-butyl-1-methyl-β-carboline-7-ol **7 [32]** were synthesized according to published procedures.

### 3.2. Synthesis of 1-Methyl-9-(prop-2-yn-1-yl)-β-carboline (***3***)

A mixture of 1-methyl-β-carboline (**2**, 1.82 g, 10 mmol) and anhydrous DMF (60 mL) was stirred at room temperature for 0.5 h, and then 95% NaH (0.37 g, 15 mmol) and 3-bromopropyne (1.8 g, 15 mmol) were added. The mixture was stirred at room temperature for 15–45 min. After completion of the reaction as indicated by TLC, the solution was poured into H_2_O (150 mL), and extracted with ethyl acetate. The organic phase was washed with water and brine, then dried over anhydrous sodium sulfate, filtered, and evaporated. The resulting oil was crystallized from ethyl ether. White crystals of **3** were obtained (1.93 g, 88%). ^1^H NMR (400 MHz, DMSO-*d*_6_) *δ*: 8.38–8.18 (m, 2H), 8.01 (s, 1H), 7.81 (d, *J* = 7.6 Hz, 1H), 7.64 (t, *J* = 7.6 Hz, 1H), 7.32 (t, *J* = 6.8 Hz, 1H), 5.48 (s, 2H), 3.39 (d, *J* = 2.0 Hz, 1H), 3.09 (s, 3H). ^13^C NMR (100 MHz, DMSO-*d*_6_) *δ*: 142.22, 141.34, 138.80, 134.74, 129.05, 128.82, 122.07, 121.34, 120.62, 113.49, 110.86, 80.58, 76.10, 34.68, 23.10.

### 3.3. Synthesis of 9-Butyl-1-Methyl-7-(Prop-2-yn-1-yloxy)-β-Carboline (***8***)

Prepared by the same procedure as compound **3** from **7** (2.54 g, 10 mmol) and 3-bromopropyne (1.8 g, 15 mmol). White crystals of **8** were obtained (2.37 g, 81%). ^1^H NMR (400 MHz, CDCl_3_) *δ*: 8.29 (d, *J* = 5.2 Hz, 1H), 7.99 (dd, *J* = 8.1, 2.0 Hz, 1H), 7.73 (d, *J* = 5.2 Hz, 1H), 6.99 (t, *J* = 2.0 Hz, 1H), 6.95–6.92 (m, 1H), 4.84 (dd, *J* = 2.4, 1.2 Hz, 2H), 4.46 (t, *J* = 8.0 Hz, 2H), 3.02 (s, 3H), 2.57 (t, *J* = 2.4 Hz, 1H), 1.86–1.77 (m, 2H), 1.50–1.40 (m, 2H), 0.98 (t, *J* = 7.2 Hz, 3H). ^13^C NMR (100 MHz, CDCl_3_) *δ*: 158.55, 142.81, 140.73, 138.28, 135.44, 129.17, 122.39, 115.97, 112.35, 108.92, 95.16, 78.5, 75.79, 56.35, 44.77, 32.77, 23.47, 20.23, 13.92.

### 3.4. General Procedure for the Synthesis of 1,2,3-Triazolyl-β-Carboline Hybrids (***5*** and ***9***)

A 50 mL Schlenk tube was charged with Cu(II)-complex L**_1_** (0.025 mmol), arylboronic acid (5 mmol), NaN_3_ (6 mmol) and dry alcohol (30 mL). The mixture was stirred at 30 °C and monitored by TLC until the arylboronic acid was consumed. Compound **3** or **8** (2.5 mmol) was added, and the solution was continuously heated at 50 °C for 2 h. After completion of the reaction, water was added to the reaction mixture, and the compound was extracted with ethyl acetate (3 × 100 mL). The organic phase was washed with water and brine, dried over anhydrous Na_2_SO_4_, and the solvent was removed under reduced pressure. The crude product was purified by flash column chromatograph on silica gel (ethyl acetate/petroleum ether as the eluent) to obtain the target products.

*1-Methyl-9-((1-phenyl-1H-1,2,3-triazol-4-yl)methyl)-β-carboline* (**5a**): White crystals (0.71g, 84%) were obtained. ^1^H NMR (400 MHz, DMSO-*d*_6_) *δ*: 8.79 (s, 1H), 8.24–8.27 (m, 2H), 8.02 (d, *J* = 5.2 Hz, 1H), 7.81–7.88 (m, 3H), 7.58–7.62 (m, 1H), 7.52–7.56 (m, 2H), 7.43–7.47 (m, 1H), 7.29 (t, *J* = 7.2 Hz, 1H), 5.99 (s, 2H), 3.13 (s, 3H). ^13^C NMR (100 MHz, DMSO-*d*_6_) *δ*: 145.53, 142.30, 141.46, 138.38, 136.89, 135.09, 130.25, 129.16, 128.67, 128.65, 121.94, 121.67, 121.38, 120.57, 120.34, 113.46, 111.18, 39.92, 23.89. HRMS calcd for C_21_H_18_N_5_ [M + H]^+^ 340.1557, found 340.1569.

*1-Methyl-9-((1-(4-(trifluoromethyl)phenyl)-1H-1,2,3-triazol-4-yl)methyl)-β-carboline* (**5b**): Slightly brown crystals (0.87 g, 86%) were obtained. ^1^H NMR (400 MHz, DMSO-*d_6_*) *δ*: 8.93 (s, 1H), 8.26 (d, *J* = 7.6 Hz, 2H), 8.10 (d, *J* = 8.4 Hz, 2H), 8.03 (d, *J* = 5.2 Hz, 1H), 7.93 (d, *J* = 8.8 Hz, 2H), 7.86 (d, *J* = 8.4 Hz, 1H), 7.58–7.63 (m, 1H), 7.30 (t, *J* = 8.0 Hz, 1H), 6.02 (s, 2H), 3.12 (s, 3H). ^13^C NMR (100 MHz, DMSO-*d*_6_) *δ*: 145.99, 142.27, 141.46, 135.09, 139.66 (q, *J* = 1.4 Hz), 138.41, 128.96 (q, *J* = 32.1 Hz), 128.71, 128.63, 127.55 (q, *J* = 3.7 Hz), 124.24 (q, *J* = 270.5 Hz), 121.95, 121.39, 121.03, 120.37, 113.47, 111.15, 23.86. ^19^F NMR (376 MHz, DMSO-*d*_6_) *δ*: −61.04. HRMS calcd for C_22_H_17_F_3_N_5_ [M + H]^+^ 408.1431, found 408.1422.

*1-Methyl-9-((1-(3,4,5-trifluorophenyl)-1H-1,2,3-triazol-4-yl)methyl)-β-carboline* (**5c**): Slightly brown crystals (0.79 g, 80%) were obtained. ^1^H NMR (400 MHz, DMSO-*d*_6_) *δ*: 8.78 (s, 1H), 8.26 (d, *J* = 6.0 Hz, 2H), 8.04 (d, *J* = 5.2 Hz, 1H), 7.98 (dd, *J* = 8.8, 6.0 Hz, 2H), 7.83 (d, *J* = 8.4 Hz, 1H), 7.58–7.62 (m, 1H), 7.30 (t, *J* = 8.0 Hz, 1H), 6.01 (s, 2H), 3.09 (s, 3H). ^13^C NMR (100 MHz, DMSO-*d*_6_) *δ*: 150.43 (ddd, *J* = 240.5, 10.1, 5.6 Hz), 145.61, 141.61, 140.89, 138.53 (dt, *J* = 249, 14.9 Hz), 137.81, 134.44, 131.92 (td, *J* = 11.5, 4.3 Hz), 128.20, 128.15, 121.52, 121.40, 120.80, 119.84, 112.93, 110.55, 105.71 (m), 23.18. ^19^F NMR (376 MHz, DMSO) *δ*: −132.58 (d, *J* = 21.8 Hz), −161.09 (t, *J* = 21.8 Hz). HRMS calcd for C_21_H_15_F_3_N_5_ [M + H]^+^ 394.1274, found 394.1288.

*1-Methyl-9-((1-(pyridin-4-yl)-1H-1,2,3-triazol-4-yl)methyl)-β-carboline* (**5d**): Slightly yellow crystals (0.61 g, 72%) were obtained. ^1^H NMR (400 MHz, Methanol-*d*_4_) *δ*: 8.66 (d, *J* = 5.6 Hz, 2H), 8.55 (s, 1H), 8.23–8.20 (m, 2H), 8.01 (d, *J* = 5.2 Hz, 1H), 7.89–7.88 (m, 2H), 7.74 (dt, *J* = 8.4, 0.8 Hz, 1H), 7.66–7.62 (m, 1H), 7.35–7.31 (m, 1H), 6.05 (s, 2H), 3.13 (s, 3H). ^13^C NMR (100 MHz, Methanol-*d*_4_) *δ*: 150.77, 146.20, 143.55, 141.82, 141.61, 137.19, 129.92, 128.60, 121.32, 121.27, 120.33, 120.26, 113.92, 113.08, 109.94, 39.85, 21.46. HRMS calcd for C_20_H_17_N_6_ [M + H]^+^ 341.1509, found 341.1498.

*1-Methyl-9-((1-(4-(9H-carbazol-9-yl)phenyl)-1H-1,2,3-triazol-4-yl)methyl)-β-carboline* (**5e**): Slightly brown crystals (0.73 g, 58%) were obtained. ^1^H NMR (400 MHz, Pyridine-*d_5_*) *δ*: 8.67 (s, 1H), 8.59 (d, *J* = 5.2 Hz, 1H), 8.30–8.26 (m, 3H), 8.05 (d, *J* = 8.8 Hz, 2H), 8.00–7.95 (m, 2H), 7.68–7.64 (m, 5H), 7.49 (s, 2H), 7.41–7.36 (m, 3H), 6.18 (s, 2H), 3.36 (s, 3H). ^13^C NMR (100 MHz, Pyridine-*d*_5_) *δ*: 146.26, 142.22, 141.62, 140.61, 138.90, 137.55, 135.71, 135.32, 129.16, 128.49, 127.86, 126.45, 123.75, 121.97, 121.91, 121.74, 120.86, 120.66, 120.64, 120.35, 113.11, 110.53, 109.88, 40.75, 23.73. HRMS calcd for C_33_H_25_N_6_ [M + H]^+^ 505.2135, found 505.2145.

*1-Methyl-9-((1-(4-ethoxycarbonyl)phenyl-1H-1,2,3-triazol-4-yl)methyl)-β-carboline* (**5f**): Yellow crystals (0.82 g, 80%) were obtained. ^1^H NMR (400 MHz, Methanol-*d*_4_) *δ*: 8.44 (s, 1H), 8.23–8.20 (m, 2H), 8.16–8.13 (m, 2H), 8.02 (d, *J* = 5.6 Hz, 1H), 7.92–7.89 (m, 2H), 7.76 (d, *J* = 8.4 Hz, 1H), 7.66–7.62 (m, 1H), 7.35–7.31 (m, 1H), 6.05 (s, 2H), 4.39 (q, *J* = 7.2 Hz, 2H), 3.15 (s, 3H), 1.40 (t, *J* = 7.2 Hz, 3H). ^13^C NMR (100 MHz, Methanol-*d*_4_) *δ*: 165.38, 145.83, 141.88, 139.96, 137.15, 137.14, 130.73, 130.43, 129.93, 128.60, 121.33, 121.27, 120.57, 120.24, 119.76, 109.98, 61.08, 39.90, 21.49, 13.13. HRMS calcd for C_24_H_22_N_5_O_2_ [M + H]^+^ 412.1768, found 412.1759.

*1-Methyl-9-((1-(4-vinylphenyl)-1H-1,2,3-triazol-4-yl)methyl)-β-carboline* (**5g**): Brown crystals (0.81 g, 89%) were obtained. ^1^H NMR (400 MHz, Methanol-*d*_4_) *δ*: 8.32 (s, 1H), 8.25 (s, 1H), 8.22(d, *J* = 8.0 Hz, 1H), 8.03 (d, *J* = 5.2 Hz, 1H), 7.76 (d, *J* = 8.4 Hz, 1H), 7.72–7.70 (m, 2H), 7.66–7.62 (m, 1H), 7.58–7.56 (m, 2H), 7.35–7.31 (m, 1H), 6.77 (dd, *J* = 17.6, 10.8 Hz, 1H), 6.04 (s, 2H), 5.85 (d, *J* = 17.6 Hz, 1H), 5.32 (d, *J* = 10.8 Hz, 1H), 3.15 (s, 3H). ^13^C NMR (100 MHz, Methanol-*d*_4_) *δ*: 145.47, 141.90, 138.37, 135.99, 135.34, 129.88, 128.58, 127.05, 121.31, 121.25, 120.39, 120.23, 120.21, 120.20, 114.39, 109.99, 56.92, 39.91, 16.96. HRMS calcd for C_23_H_20_N_5_ [M + H]^+^ 366.1713, found 366.1720.

*1-Methyl-9-((1-(4-(trifluoromethoxy)phenyl)-1H-1,2,3-triazol-4-yl)methyl)-β-carboline* (**5h**): Slightly brown crystals (0.84 g, 79%) were obtained. ^1^H NMR (400 MHz, Methanol-*d*_4_) *δ*: 6.83 (s, 1H), 6.69–6.66 (m, 2H), 6.47 (d, *J* = 5.6 Hz, 1H), 6.35–6.31 (m, 2H), 6.21 (d, *J* = 8.4 Hz, 1H), 6.12–6.08 (m, 1H), 5.89 (d, *J* = 8.4 Hz, 2H), 5.81–5.77 (m, 1H), 4.50 (s, 2H), 1.60 (s, 3H). ^13^C NMR (100 MHz, Methanol-*d*_4_) *δ*: 144.21, 140.35, 140.10, 135.61, 133.97, 133.57, 128.40, 127.06, 120.47, 120.40, 119.79, 119.74, 119.18, 118.70, 111.55, 108.44, 38.37, 19.94. ^19^F NMR (376 MHz, DMSO-*d*_6_) *δ*: −59.68. HRMS calcd for C_22_H_17_F_3_N_5_O [M + H]^+^ 424.1380, found 424.1388.

*1-methyl-9-((1-(4-methoxyphenyl)-1H-1,2,3-triazol-4-yl)methyl)-β-carboline* (**5i**): Brown crystals (0.84 g, 91%) were obtained. ^1^H NMR (400 MHz, Methanol-*d*_4_) *δ*: 8.24–8.21 (m, 3H), 8.02 (d, *J* = 5.2 Hz, 1H), 7.75 (d, *J* = 8.4 Hz, 1H), 7.66–7.60 (m, 3H), 7.35–7.31 (m, 1H), 7.05–7.01 (m, 2H), 6.02 (s, 2H), 3.83 (s, 3H), 3.14 (s, 3H). ^13^C NMR (100 MHz, Methanol-*d*_4_) *δ*: 160.17, 145.23, 141.89, 130.10, 129.87, 128.58, 121.87, 121.86, 121.29, 121.24, 120.65, 120.20, 114.39, 110.00, 54.68, 39.90, 16.97. HRMS calcd for C_22_H_20_N_5_O [M + H]^+^ 370.1662, found 370.1669.

*1-Methyl-9-((1-(p-tolyl)-1H-1,2,3-triazol-4-yl)methyl)-β-carboline* (**5j**): Slightly brown crystals (0.76 g, 86%) were obtained. ^1^H NMR (400 MHz, Methanol-*d*_4_) *δ*: 8.36 (s, 1H), 8.26 (d, *J* = 8.0 Hz, 2H), 8.17 (d, *J* = 5.6 Hz, 1H), 7.81 (d, *J* = 8.4 Hz, 1H), 7.72–7.68 (m, 1H), 7.59 (d, *J* = 8.4 Hz, 2H), 7.38 (t, *J* = 7.6 Hz, 1H), 7.30 (d, *J* = 8.4 Hz, 2H), 6.03 (s, 2H), 3.22 (s, 3H), 2.37 (s, 3H). ^13^C NMR (100 MHz, Methanol-*d*_4_) *δ*: 144.41, 143.31, 139.19, 134.49, 130.57, 129.89, 122.18, 121.40, 120.82, 120.55, 120.08, 114.41, 110.52, 39.87, 19.60. HRMS calcd for C_22_H_20_N_5_ [M + H]^+^ 354.1713, found 354.1703.

*1-Methyl-9-((1-(4-fluorophenyl)-1H-1,2,3-triazol-4-yl)methyl)-β-carboline* (**5k**): Slightly brown crystals (0.71 g, 80%) were obtained. ^1^H NMR (400 MHz, DMSO-*d*_6_) *δ*: 8.82 (s, 1H), 8.27–8.24 (m, 2H), 8.03 (d, *J* = 5.2 Hz, 1H), 7.90–7.86 (m, 3H), 7.63–7.58 (m, 1H), 7.43–7.38 (m, 2H), 7.29 (t, *J* = 7.6 Hz, 1H), 6.00 (s, 2H), 3.13 (s, 3H). ^13^C NMR (100 MHz, DMSO-*d*_6_) *δ*: 162.04 (d, *J* = 244 Hz), 145.48, 142.33, 141.41, 138.34, 135.09, 133.43 (d, *J* = 2.6 Hz), 128.70, 128.64, 122.97, 122.88, 121.99 (d, *J* = 10 Hz), 121.33, 120.35, 117.11 (d, *J* = 23.1 Hz), 113.50, 111.20, 23.88. HRMS calcd for C_21_H_17_FN_5_ [M + H]^+^ 358.1463, found 358.1470.

*9-Butyl-1-methyl-7-((1-(4-(trifluoromethyl)phenyl)-1H-1,2,3-triazol-4-yl)methoxy)-β-carboline* (**9a**): Slightly yellow crystals (0.41 g, 85%) were obtained. ^1^H NMR (400 MHz, DMSO-*d*_6_) *δ*: 9.17 (s, 1H), 8.21–8.17 (m, 3H), 8.12 (d, *J* = 8.4 Hz, 1H), 8.01 (d, *J* = 8.4 Hz, 2H), 7.88 (d, *J* = 5.2 Hz, 1H), 7.40 (d, *J* = 2.4 Hz, 1H), 6.98 (dd, *J* = 8.8, 2.0 Hz, 1H), 5.45 (s, 2H), 4.56 (t, *J* = 7.6 Hz, 2H), 2.95 (s, 3H), 1.75–1.67 (m, 2H), 1.42–1.32 (m, 2H), 0.89 (t, *J* = 7.6 Hz, 3H). ^13^C NMR (100 MHz, DMSO-*d*_6_) *δ*: 159.49, 144.80, 143.10, 141.07, 139.80 (q, *J* = 1.5 Hz), 138.22, 135.10, 129.40 (q, *J* = 32.4 Hz), 128.79, 127.73 (q, *J* = 3.6 Hz), 124.27 (q, *J* = 270.5 Hz) 123.63, 122.95, 121.04, 115.06, 112.76, 109.96, 95.37, 61.99, 44.38, 32.93, 23.55, 19.98, 14.19. HRMS calcd for C_26_H_25_F_3_N_5_O [M + H]^+^ 480.2017, found 480.2009.

*9-Butyl-1-methyl-7-((1-(3,4,5-trifluorophenyl)-1H-1,2,3-triazol-4-yl)methoxy)-β-carboline* (**9b**): Slightly brown crystals (0.43 g, 91%) were obtained. ^1^H NMR (400 MHz, DMSO-*d*_6_ ) *δ*: 8.76 (s, 1H), 8.18–8.13 (m, 2H), 8.04 (s, 1H), 7.86–7.82 (m, 2H), 7.32 (s, 1H), 7.07 (d, *J* = 8.8 Hz, 1H), 5.47 (s, 2H), 4.63 (t, *J* = 7.6 Hz, 2H), 3.07 (s, 3H), 1.87–1.79 (m, 2H), 1.52–1.43 (m, 2H), 1.00 (t, *J* = 7.6 Hz, 3H). ^13^C NMR (100 MHz, DMSO-*d*_6_) *δ*: 159.54, 152.34 (ddd, *J* = 246.6, 10.0, 5.2 Hz), 144.86, 143.17, 140.91, 138.70 (dt, *J* = 250, 18 Hz), 137.90, 135.02, 132.58 (td, *J* = 12.1, 3.6 Hz), 128.92, 123.64, 122.96, 115.02, 112.78, 110.00, 106.17 (m), 95.29, 62.01, 44.38, 32.91, 23.32, 19.97, 14.16. HRMS calcd for C_25_H_23_F_3_N_5_O [M + H]^+^ 466.1849, found 466.1860.

*9-Butyl-1-methyl-7-((1-(4-(trifluoromethoxy)phenyl)-1H-1,2,3-triazol-4-yl)methoxy)-β-carboline* (**9c**): Yellow crystals (0.44 g, 88%) were obtained. ^1^H NMR (400 MHz, DMSO-*d*_6_) *δ*: 9.10 (s, 1H), 8.17 (d, *J* = 5.2 Hz, 1H), 8.15–8.04 (m, 3H), 7.89 (d, *J* = 5.2 Hz, 1H), 7.65 (d, *J* = 8.0 Hz, 2H), 7.41 (d, *J* = 2.0 Hz, 1H), 6.97 (dd, *J* = 8.4, 2.0 Hz, 1H), 5.43 (s, 2H), 4.58 (t, *J* = 7.6 Hz, 2H), 2.95 (s, 3H), 1.74–1.66 (m, 2H), 1.41–1.32 (m, 2H), 0.89 (t, *J* = 7.6 Hz, 3H). ^13^C NMR (100 MHz, DMSO-*d*_6_) *δ*: 159.46, 148.34, 144.55, 143.11, 141.05, 138.20, 135.87, 128.79, 123.77, 123.11, 122.95, 122.57, 121.73, 119.17, 114.97, 112.77, 110.06, 95.35, 61.87, 44.34, 32.96, 23.52, 19.93, 14.18. HRMS calcd for C_26_H_25_F_3_N_5_O_2_ [M + H]^+^ 496.1955, found 496.1962.

*9-Butyl-7-((1-(4-methoxyphenyl)-1H-1,2,3-triazol-4-yl)methoxy)-1-methyl-β-carboline* (**9d**): White crystals (0.39 g, 89%) were obtained. ^1^H NMR (400 MHz, DMSO-*d*_6_) *δ*: 8.55 (s, 1H), 8.04 (d, *J* = 8.4 Hz, 1H), 7.89 (s, 1H), 7.76–7.69 (m, 2H), 7.20 (d, *J* = 2.0 Hz, 1H), 7.13–7.05 (m, 2H), 7.00 (dd, *J* = 8.4, 2.0 Hz, 1H), 5.40 (s, 2H), 4.53 (t, *J* = 7.6 Hz, 2H), 3.87 (s, 3H), 2.97 (s, 3H), 1.80–1.72 (m, 2H), 1.47–1.37 (m, 2H), 0.96 (t, *J* = 7.2 Hz, 3H). ^13^C NMR (100 MHz, DMSO-*d*_6_) *δ*: 159.77, 159.56, 144.10, 143.12, 141.08, 138.16, 130.45, 128.80, 123.35, 122.91, 122.22, 115.35, 114.97, 112.78, 109.99, 95.31, 62.02, 56.02, 44.36, 32.94, 23.57, 19.97, 14.20. HRMS calcd for C_26_H_28_N_5_O_2_ [M + H]^+^ 442.2238, found 442.2247.

*9-Butyl-1-methyl-7-((1-(p-tolyl)-1H-1,2,3-triazol-4-yl)methoxy)-β-carboline* (**9e**): Slightly yellow crystals (0.36 g, 86%) were obtained. ^1^H NMR (400 MHz, DMSO-*d*_6_) *δ*: 9.06 (s, 1H), 8.52 (d, *J* = 6.0 Hz, 1H), 8.41 (d, *J* = 8.8 Hz, 1H), 8.35 (d, *J* = 6.4 Hz, 1H), 7.81 (d, *J* = 8.4 Hz, 2H), 7.63 (d, *J* = 2.0 Hz, 1H), 7.42 (d, *J* = 8.4 Hz, 2H), 7.14 (dd, *J* = 8.8, 2.0 Hz, 1H), 5.49 (s, 2H), 4.69 (t, *J* = 8.0 Hz, 2H), 3.26 (s, 3H), 2.39 (s, 3H), 1.82–1.74 (m, 2H), 1.45–1.35 (m, 2H), 0.91 (t, *J* = 7.2 Hz, 3H). ^13^C NMR (100 MHz, DMSO-*d*_6_) *δ*: 161.94, 146.31, 143.74, 138.92, 137.71, 134.76, 133.82, 133.18, 130.74, 129.45, 125.00, 123.56, 120.48, 114.86, 113.71, 113.15, 95.24, 62.27, 44.83, 32.86, 21.06, 19.89, 17.93, 14.17. HRMS calcd for C_26_H_28_N_5_O [M + H]^+^ 426.2288, found 426.2281.

*9-Butyl-7-((1-(4-fluorophenyl)-1H-1,2,3-triazol-4-yl)methoxy)-1-methyl-β-carboline* (**9f**): White crystals (041 g, 87%) were obtained. ^1^H NMR (400 MHz, DMSO-*d*_6_) *δ*: 9.03 (s, 1H), 8.18 (d, *J* = 5.2 Hz, 1H), 8.12 (d, *J* = 8.8 Hz, 1H), 8.03–7.96 (m, 2H), 7.89 (d, *J* = 5.2 Hz, 1H), 7.48 (t, *J* = 8.8 Hz, 2H), 7.41 (d, *J* = 2.0 Hz, 1H), 6.98 (dd, *J* = 8.8, 2.0 Hz, 1H), 5.43 (s, 2H), 4.56 (t, *J* = 7.6 Hz, 2H), 2.95 (s, 3H), 1.75–1.67 (m, 2H), 1.42–1.33 (m, 2H), 0.90 (t, *J* = 7.6 Hz, 3H). ^13^C NMR (100 MHz, DMSO-*d*_6_) *δ*: 160.91(d, *J* = 245 Hz), 159.52, 144.39, 143.11, 141.05, 138.20, 135.08, 133.61 (d, *J* = 2.8 Hz), 128.80, 123.64, 122.98 (d, *J* = 8.7 Hz), 122.93, 117.22 (d, *J* = 23.3 Hz), 115.00, 112.75, 109.99, 95.33, 61.98, 44.36, 32.94, 23.54, 19.96, 14.20. HRMS calcd for C_25_H_25_FN_5_O [M + H]^+^ 430.2038, found 430.2044.

### 3.5. Biological Assays

The antifungal activity of the synthesized compounds was performed according to previously reported procedures [33]. The fungicidal activity of the target compounds against *R. solani*, *F. oxysporum*, *B. cinerea* Pers., sunflower sclerotinia rot and rape sclerotinia rot were evaluated using a mycelium growth rate test [17]. Carbendazim and azoxystrobin standard purchased from J&K Scientific Ltd. (Beijing, China), were used as a control, treating it in the same way. The relative inhibition ratio (%) was calculated using the following equation:

$$\mathrm{The}\;\mathrm{relative}\;\mathrm{inhibition}\;\mathrm{ratio}\;\left(\%\right)=\frac{\mathrm{Colony}\;\mathrm{diameter}\;\mathrm{of}\;\mathrm{control}-\;\mathrm{colony}\;\mathrm{diameter}\;\mathrm{of}\;\mathrm{treated})}{\mathrm{colony}\;\mathrm{diameter}\;\mathrm{of}\;\mathrm{control}\;\mathrm{mycelial}\;\mathrm{disk}\;\mathrm{diameter}}\times \;100\%.$$

## 4. Conclusions

In order to find potential activity from β-carboline derivatives for further structural optimization, in this study, two series of new aryl-1,2,3-triazole-β-carboline hybrids were synthesized, and first assayed for their fungicidal activities in vitro. The antifungal evaluation of the novel hybrids showed that, among the tested compounds, 1-methyl-9-((1-phenyl-1*H*-1,2,3-triazol-4-yl)methyl)-β-carboline (**5a**), 1-methyl-9-((1-(4-(trifluoromethyl)phenyl)-1*H*-1,2,3-triazol-4-yl)methyl)-β-carboline (**5b**), 1-methyl-9-((1-(3,4,5-trifluorophenyl)-1*H*-1,2,3-triazol-4-yl)methyl)-β-carboline (**5c**), and 9-butyl-1-methyl-7-((1-(3,4,5-trifluorophenyl)-1*H*-1,2,3-triazol-4-yl)methoxy)-β-carboline (**9b**) showed satisfactory antifungal activity against sunflower sclerotinia rot. Specifically, compound **9b** also exhibited high broad-spectrum fungicidal activity against all the tested fungi with inhibition rates of 58.3%, 18.52%, 63.07%, 84.47%, and 81.23%. However, for *F. oxysporum*, all the target compounds showed no in vitro antifungal activities with an inhibition rate lower than 20%.

## Figures and Tables

**Table 1:** Cu(II)-complex-catalyzed one-pot synthesis of aryl-1,2,3-triazole-β-carboline hybrids from phenylboronic acid in ethanol: optimization of the catalytic conditions.

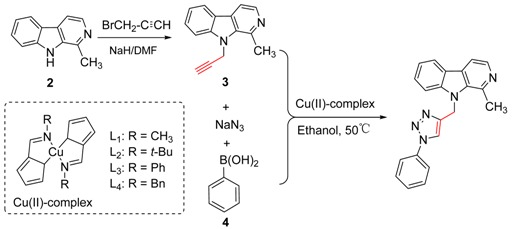			
Entry	Catalyst (mol %)	t/h	Yield/%
1	L_1_(1)	8 + 2	84
2	L_2_(1)	8 + 2	80
3	L_3_(1)	8 + 2	69
4	L_4_(1)	8 + 2	71
5	-	8 + 2	0
6	L_1_(0.5)	8 + 2	67

**Table 2:** Fungicidal activities of compounds **5a**–**k**, **9a**–**f** against four kinds of fungi (50 μg/mL) ^a^.

Componds	Inhibition Ratio (%) ^b^					ClogP ^c^
	RS	FO	BCP	SCR	RSR	
**5a**	35.57	−2.60	51.14	**85.04**	22.22	4.251
**5b**	30.68	−0.28	38.64	**86.93**	0.00	5.435
**5c**	33.52	0.65	34.85	**85.98**	53.26	4.788
**5d**	25.23	−1.67	36.36	58.14	0.00	3.468
**5e**	28.07	−2.37	15.53	71.78	17.05	8.437
**5f**	27.84	1.11	14.02	73.48	35.44	5.063
**5g**	33.52	−0.28	15.91	67.99	26.05	4.976
**5h**	35.80	−2.60	19.70	44.70	55.36	5.588
**5i**	30.45	1.81	16.29	65.34	19.54	4.479
**5j**	34.09	−3.76	7.39	67.80	35.25	4.750
**5k**	34.09	0.19	5.11	70.08	0.00	4.566
**9a**	36.59	−0.97	12.69	40.53	10.76	7.306
**9b**	**58.30**	**18.52**	**63.07**	**84.47**	**81.23**	6.659
**9c**	39.20	7.85	16.86	79.31	52.11	7.459
**9d**	28.75	2.27	**63.45**	76.70	47.31	6.350
**9e**	47.72	−7.24	28.03	44.51	28.91	6.621
**9f**	35.34	0.65	24.43	75.57	19.28	6.437
carbendazim	**81.82**	70.98	**88.07**	**89.77**	**100**	
azoxystrobin	**54.55**	51.25	**83.71**	**88.07**	**88.51**	

^a^ RS, *R. solani*; FO, *F. oxysporum*; BCP, *B. cinerea* Pers.; SCR, sunflower sclerotinia rot; RSR, rape sclerotinia rot. The data in bold are used to emphasize that these compounds showed good activity. ^b^ significant inhibitory effect: inhibitory rate ≥ 80%, moderate: inhibition rate ranges from 50% to 80%, weak: inhibition rate ranges from 20% to 50%. ^c^ ClogP represent the calculated *n*-octanol/water partition coefficient (log Pow), and the values produced by Chemdraw software.

## Supplementary Materials

The following are available online, ^1^H and ^13^C NMR spectra for the target compounds are available online.
