# Supplementary material for: Design, Synthesis, and Antifungal Activity of Novel Aryl-1,2,3-Triazole-β-Carboline Hybrids
Source: Molecules. 2018 Jun 4;23(6):1344. doi: 10.3390/molecules23061344 (PMC6100246; doi:10.3390/molecules23061344)
Supplement: Supplementary file 1 [file molecules-23-01344-s001.pdf]

Supporting Information

# Synthesis, antifungal activity and structure-activity relationships of novel aryl-1,2,3-triazole-β-carboline hybrids

**Xin-Yu Huo [1,†], Liang Guo [1,†], Xiao-Fei Chen [1], Yue-ting Zhou[2], Jie Zhang [1,*], Xiao-Qiang Han[2,*] and Bin Dai [1,*]**

[1] School of Chemistry and Chemical Engineering, Key Laboratory for Green Processing of Chemical Engineering of Xinjiang Bingtuan, Shihezi University, Shihezi 832003, China;

[2] College of Agricultural, Key Laboratory at Universities of Xinjiang Uygur Autonomous Region for Oasis Agricultural Pest Management and Plant Protection Resource Utilization, Shihezi University, Shihezi, 832003, China;

## Contents

1. **Figure S1** the $^{1}H$ NMR and $^{13}C$ NMR spectrum of compound **4a** ·········· S2
2. **Figure S2** the $^{1}H$ NMR and $^{19}F$ NMR spectrum of compound **4b** ·········· S3
3. **Figure S3** the $^{1}H$ NMR and $^{13}C$ NMR spectrum of compound **4c** ·········· S4
4. **Figure S4** the $^{1}H$ NMR and $^{13}C$ NMR spectrum of compound **4d** ·········· S5
5. **Figure S5** the $^{1}H$ NMR and $^{13}C$ NMR spectrum of compound **4e** ·········· S6
6. **Figure S6** the $^{1}H$ NMR and $^{13}C$ NMR spectrum of compound **4f** ·········· S7
7. **Figure S7** the $^{1}H$ NMR and $^{13}C$ NMR spectrum of compound **4g** ········· S8
8. **Figure S8** the $^{1}H$ NMR and $^{13}C$ NMR spectrum of compound **4h** ········· S9
9. **Figure S9** the $^{1}H$ NMR and $^{13}C$ NMR spectrum of compound **4i** ········· S10
10. **Figure S10** the $^{1}H$ NMR and $^{13}C$ NMR spectrum of compound **4j** ······ S11
11. **Figure S11** the $^{1}H$ NMR and $^{13}C$ NMR spectrum of compound **4k** ······ S12
12. **Figure S12** the $^{1}H$ NMR and $^{13}C$ NMR spectrum of compound **8a** ······ S13
13. **Figure S13** the $^{1}H$ NMR and $^{13}C$ NMR spectrum of compound **8b** ······ S14
14. **Figure S14** the $^{1}H$ NMR and $^{13}C$ NMR spectrum of compound **8c** ······ S15
15. **Figure S15** the $^{1}H$ NMR and $^{13}C$ NMR spectrum of compound **8d** ······ S16
16. **Figure S16** the $^{1}H$ NMR and $^{13}C$ NMR spectrum of compound **8e** ······· S17
17. **Figure S17** the $^{1}H$ NMR and $^{13}C$ NMR spectrum of compound **8f** ······· S18

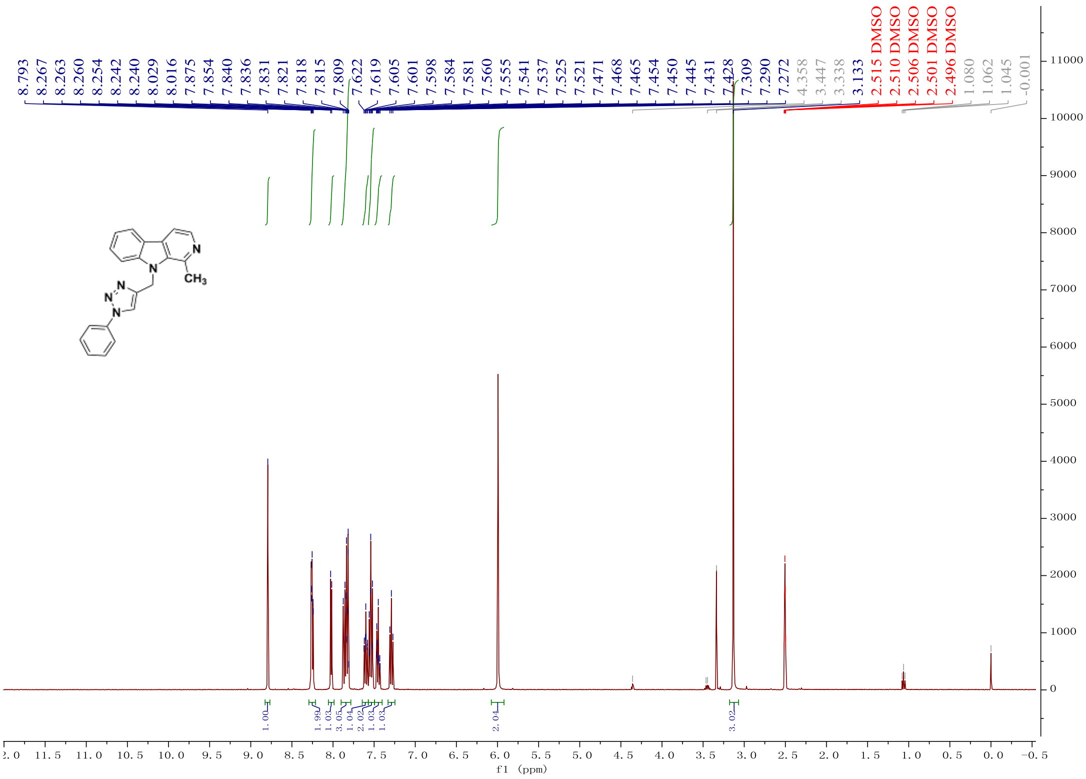


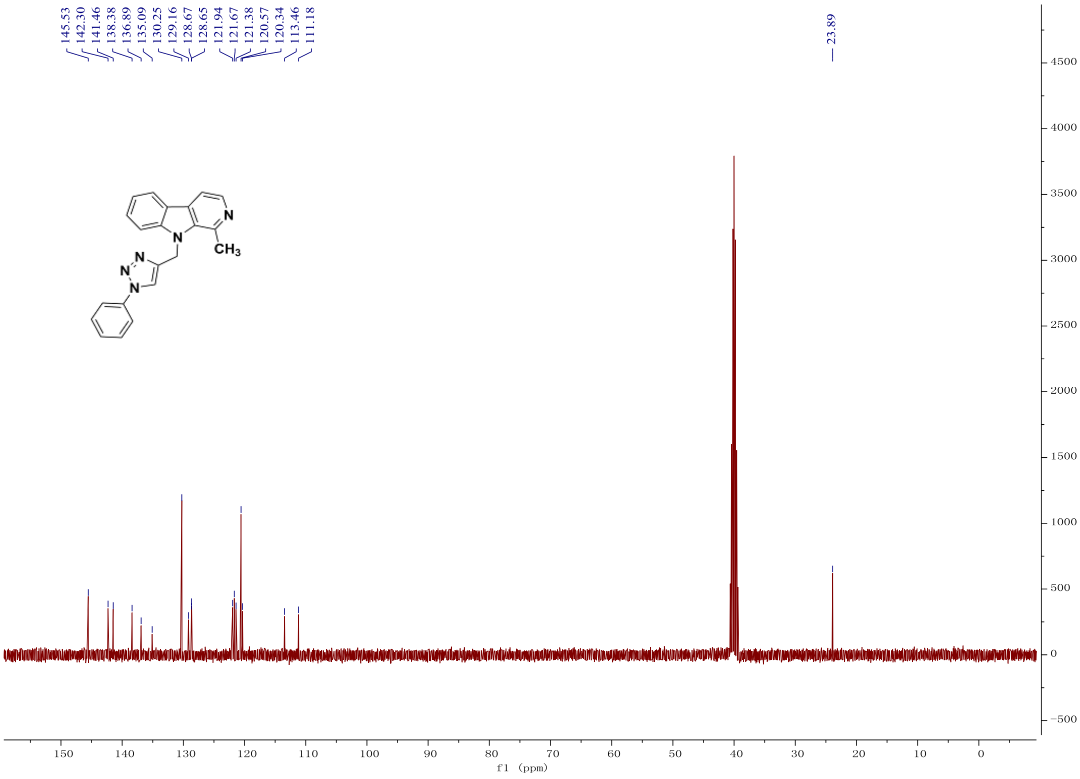


**Figure S1** the $^{1}$H NMR spectrum and $^{13}$C NMR spectrum of compound **4a**

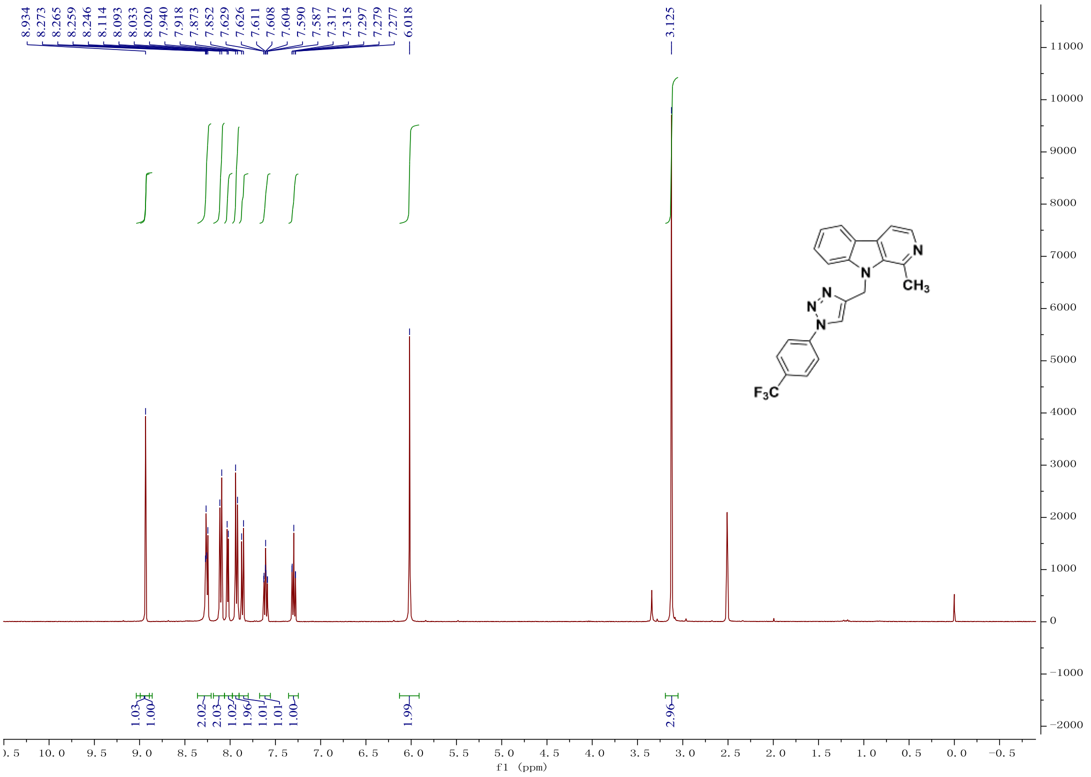


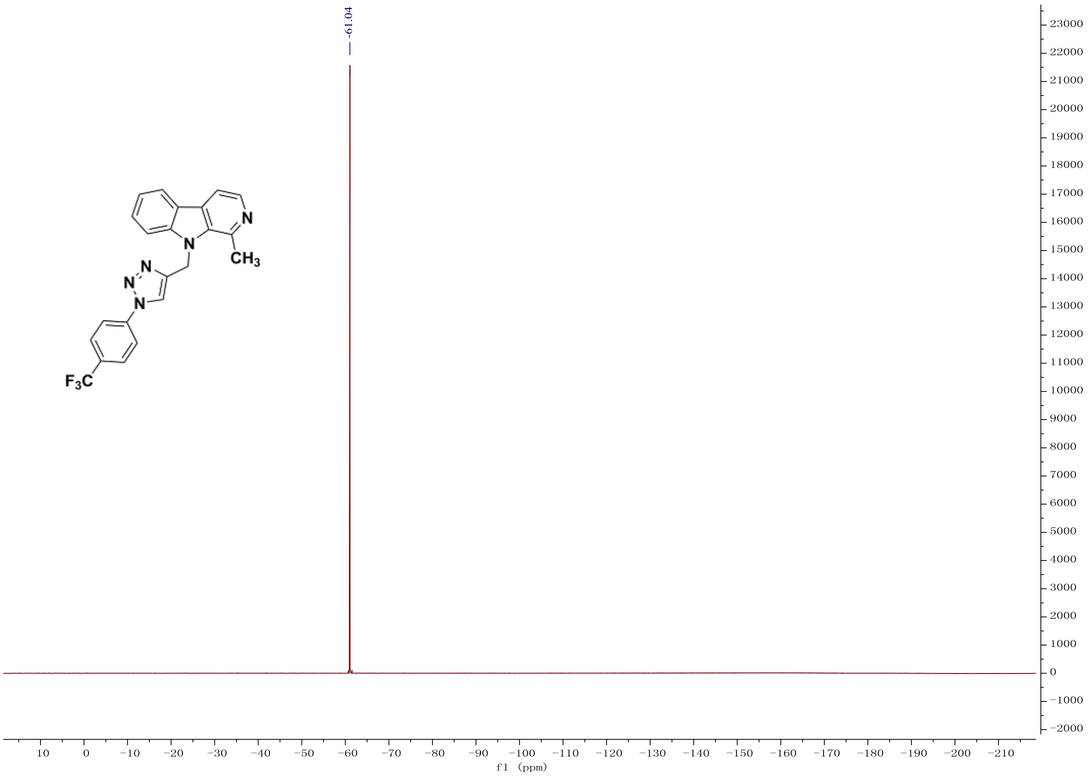


**Figure S2** the $^{1}H$ NMR spectrum and $^{19}F$ NMR spectrum of compound **4b**

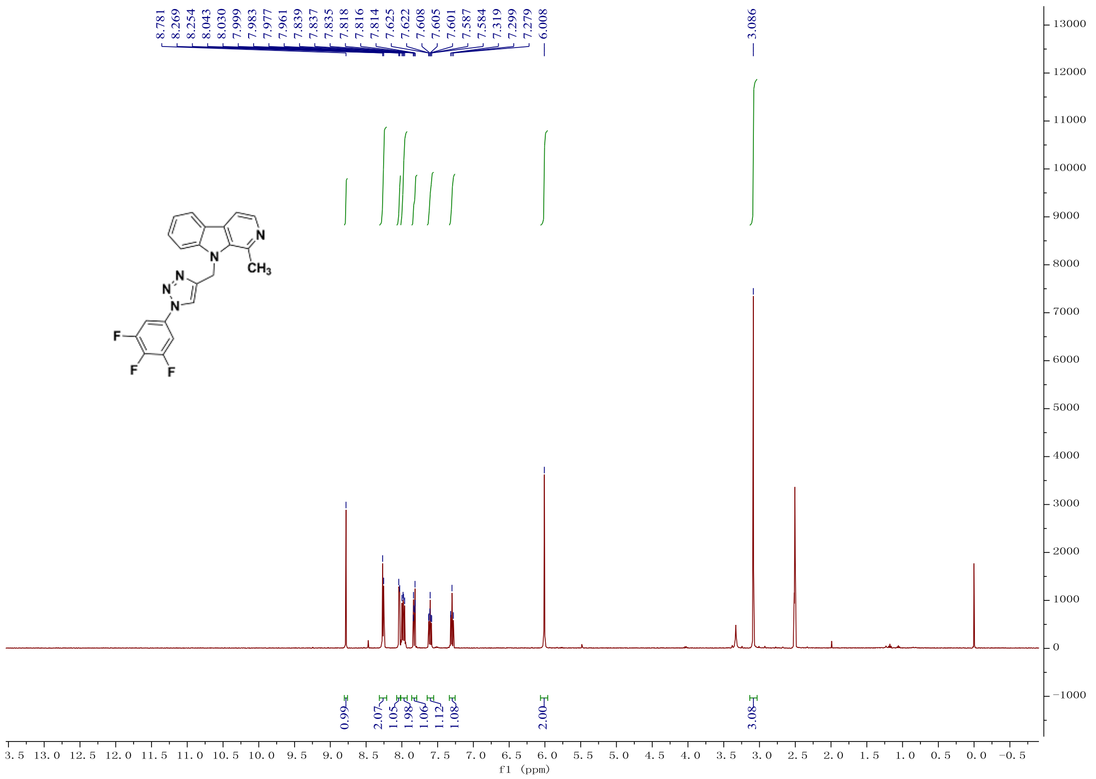

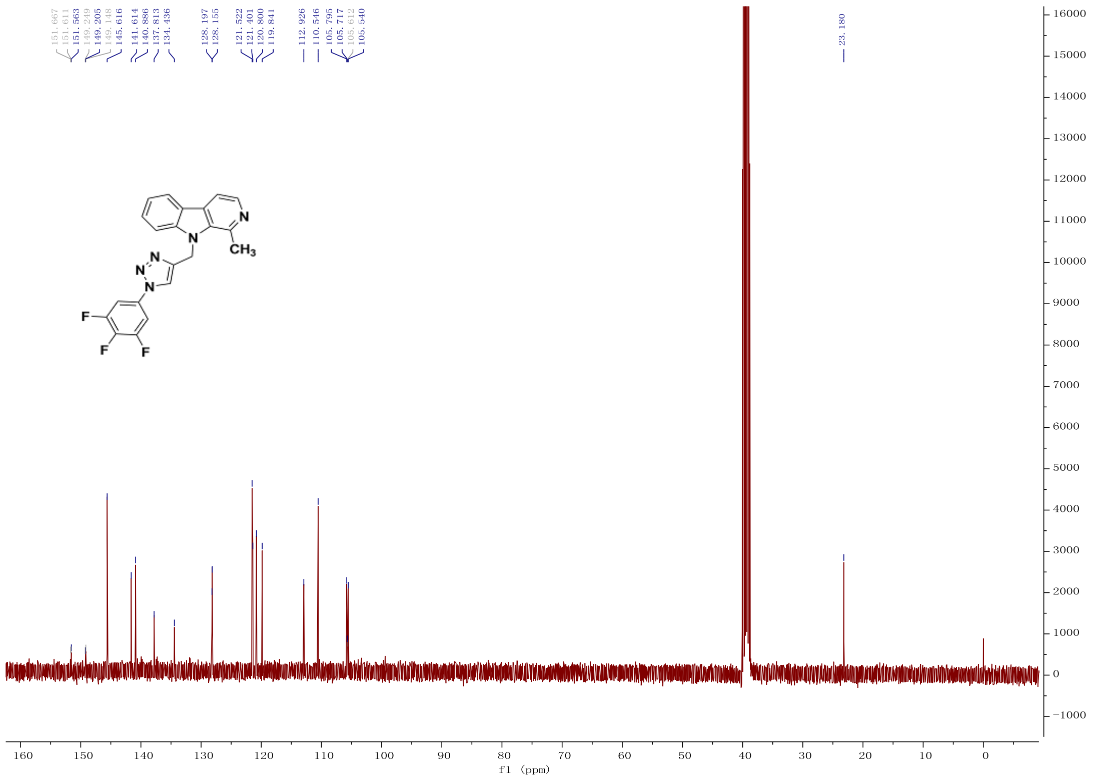

**Figure S3** the $^{1}H$ NMR spectrum and $^{13}C$ NMR spectrum of compound **4c**

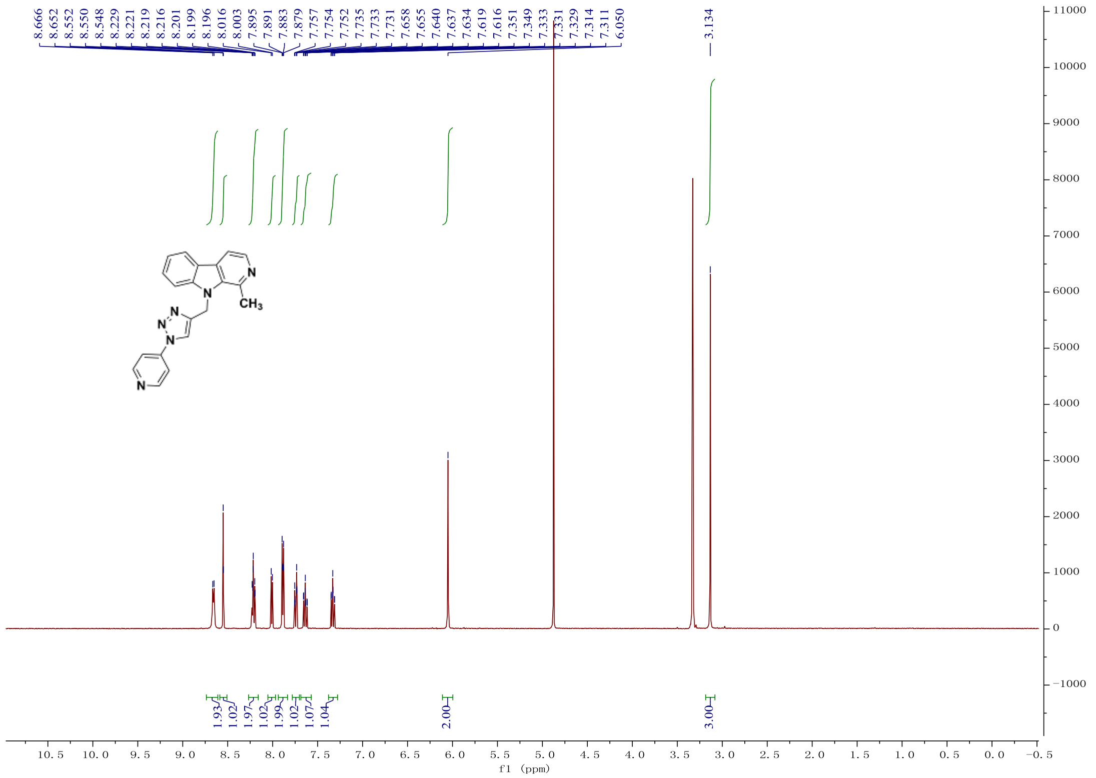


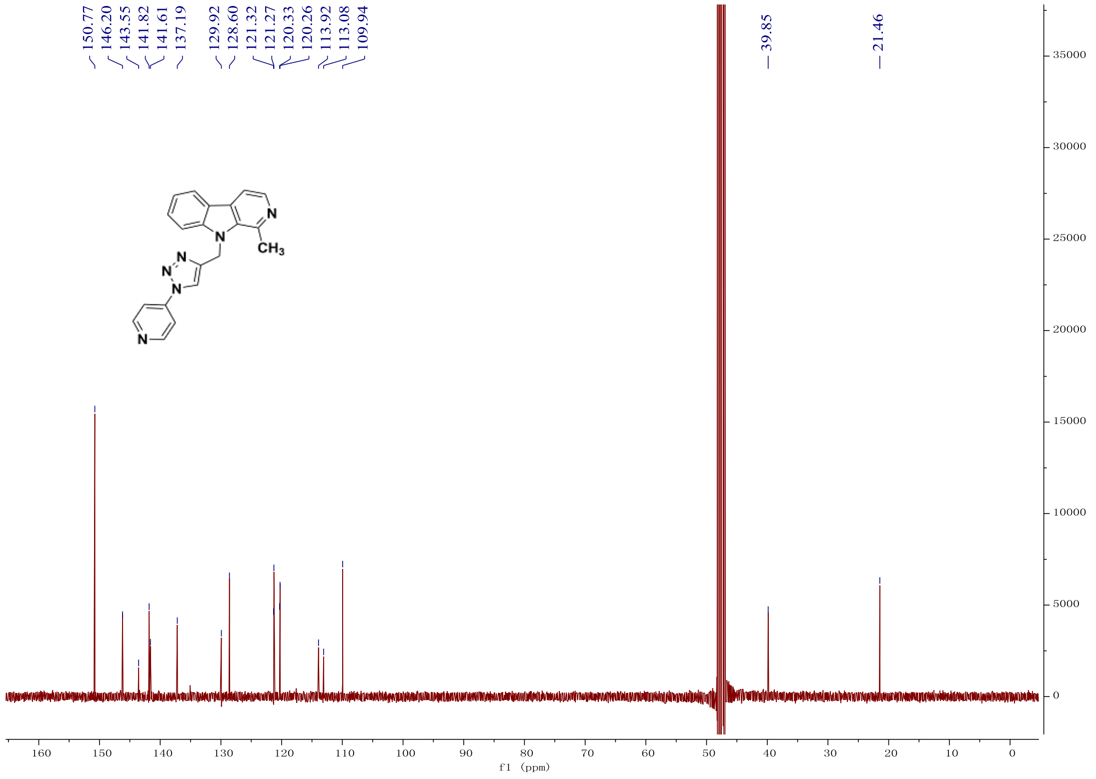


**Figure S4** the $^{1}$H NMR spectrum and $^{13}$C NMR spectrum of compound **4d**

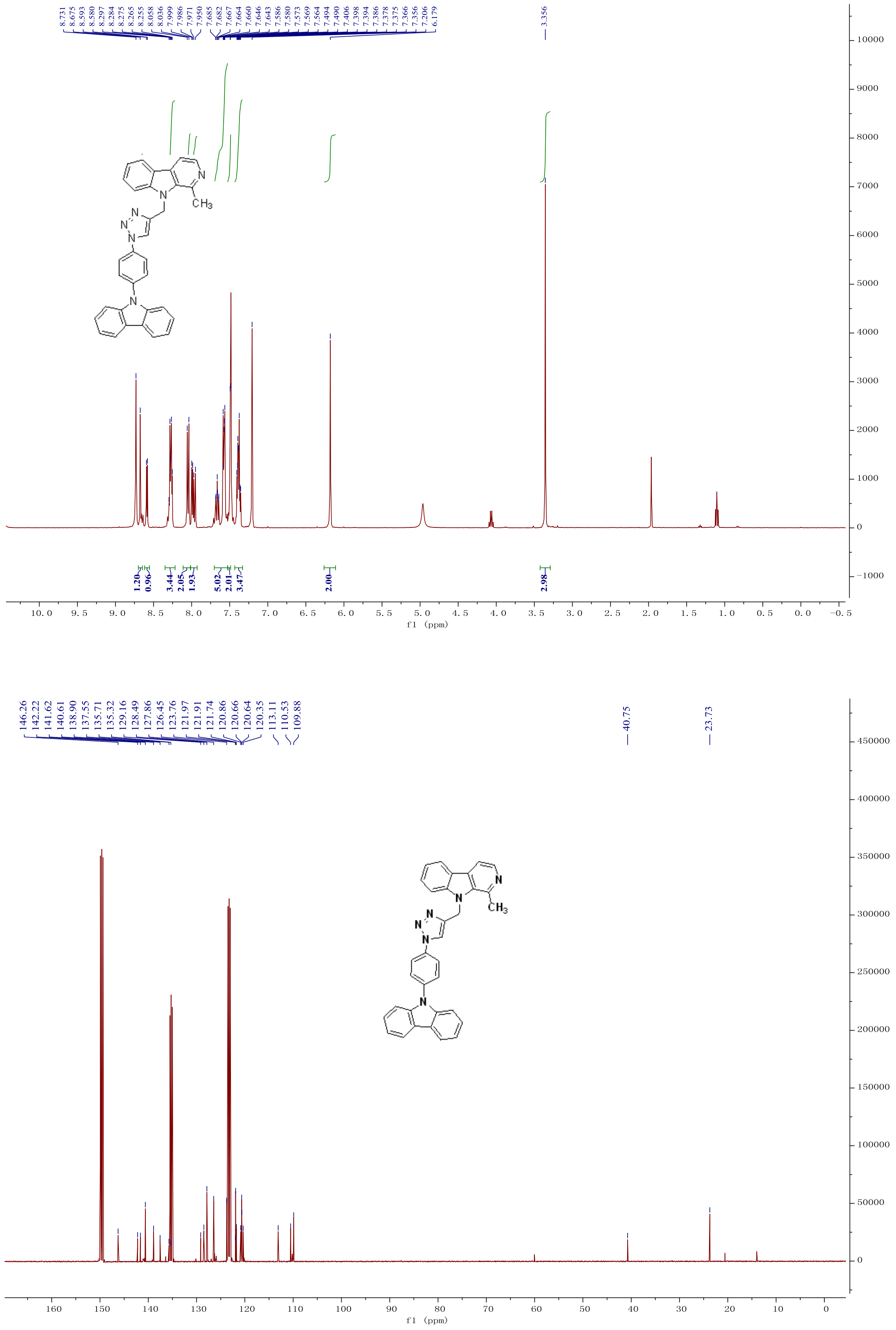


**Figure S5** the $^{1}H$ NMR spectrum and $^{13}C$ NMR spectrum of compound **4e**

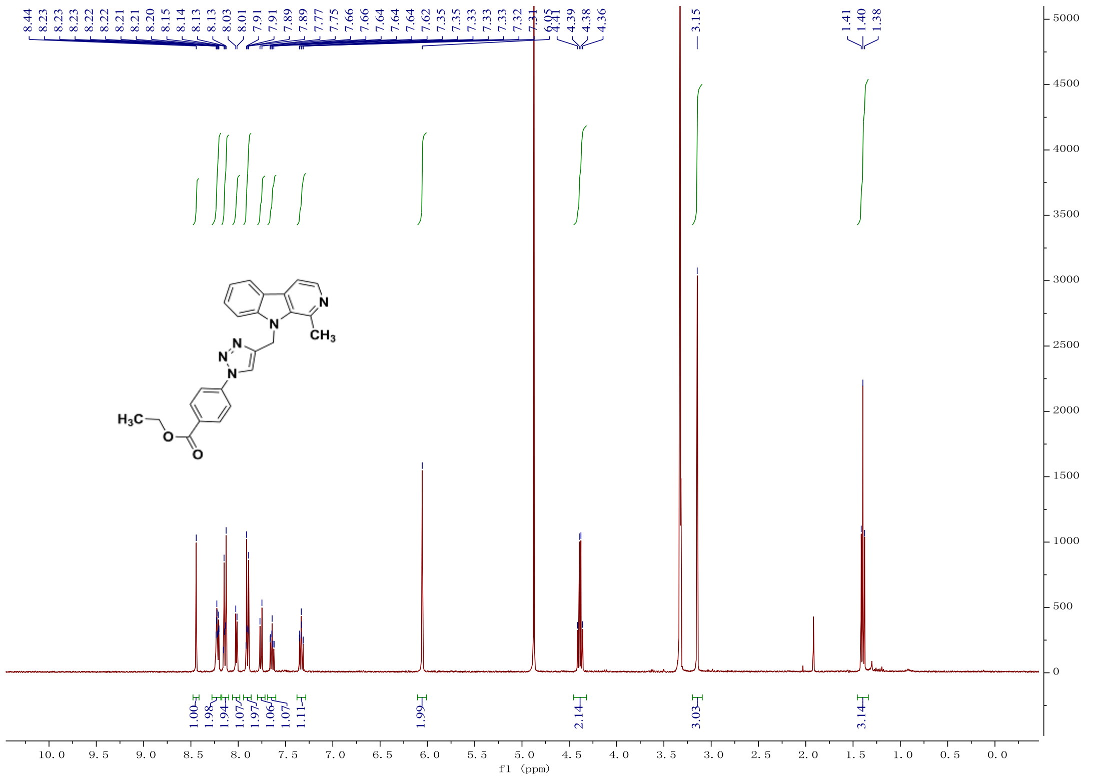


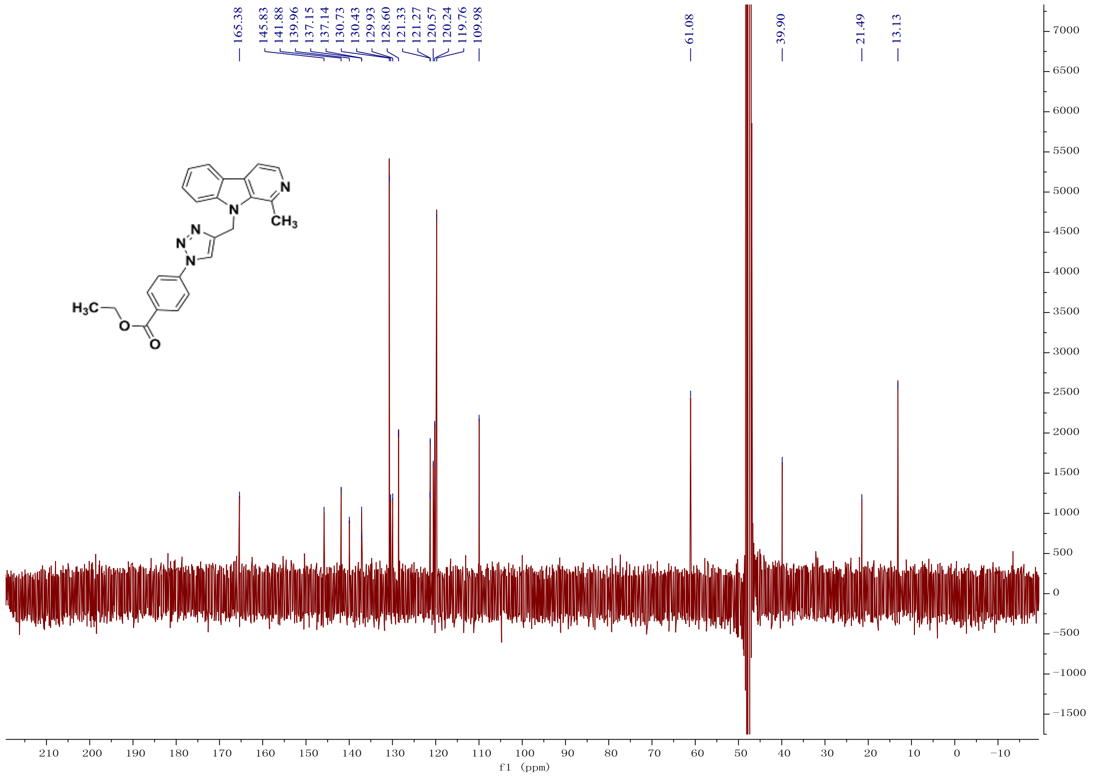


**Figure S6** the $^{1}$H NMR spectrum and $^{13}$C NMR spectrum of compound **4f**

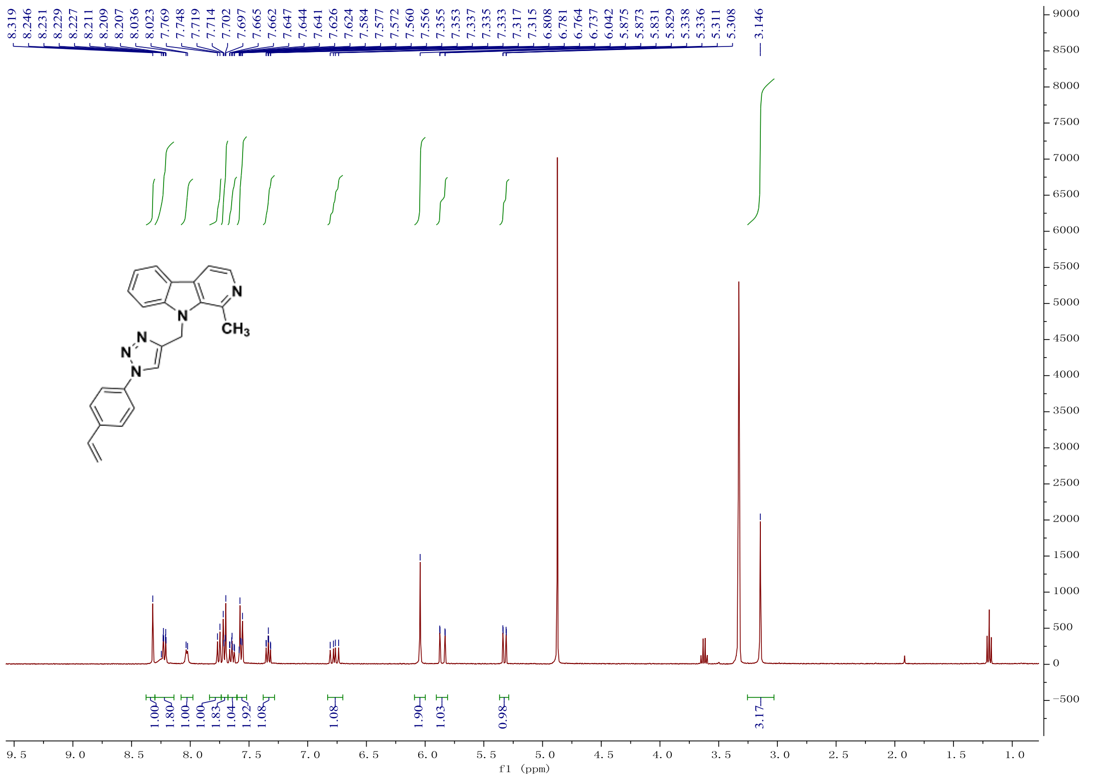

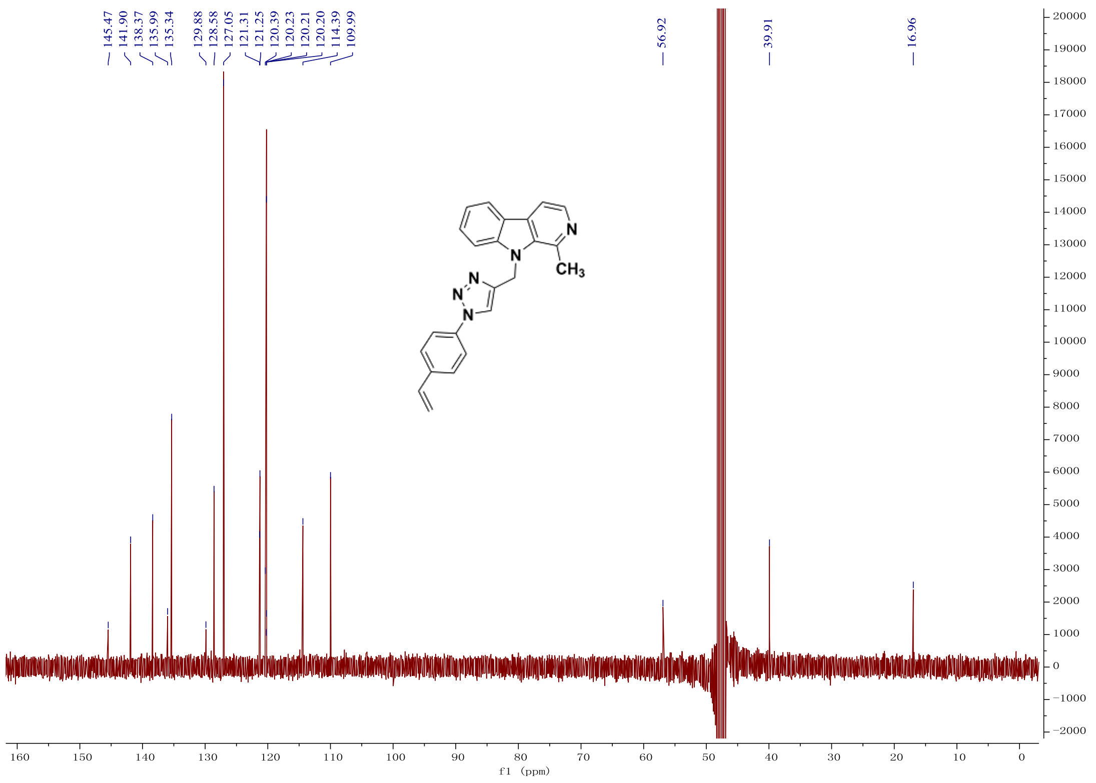

**Figure S7** the $^{1}H$ NMR spectrum and $^{13}C$ NMR spectrum of compound **4g**

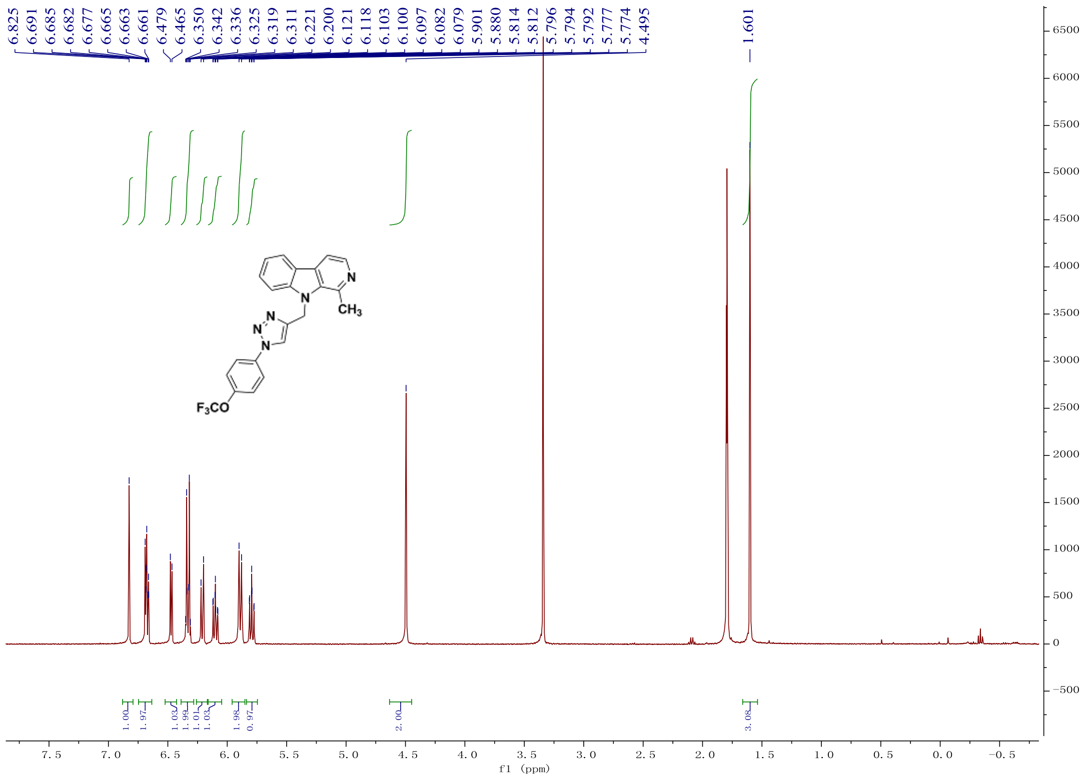


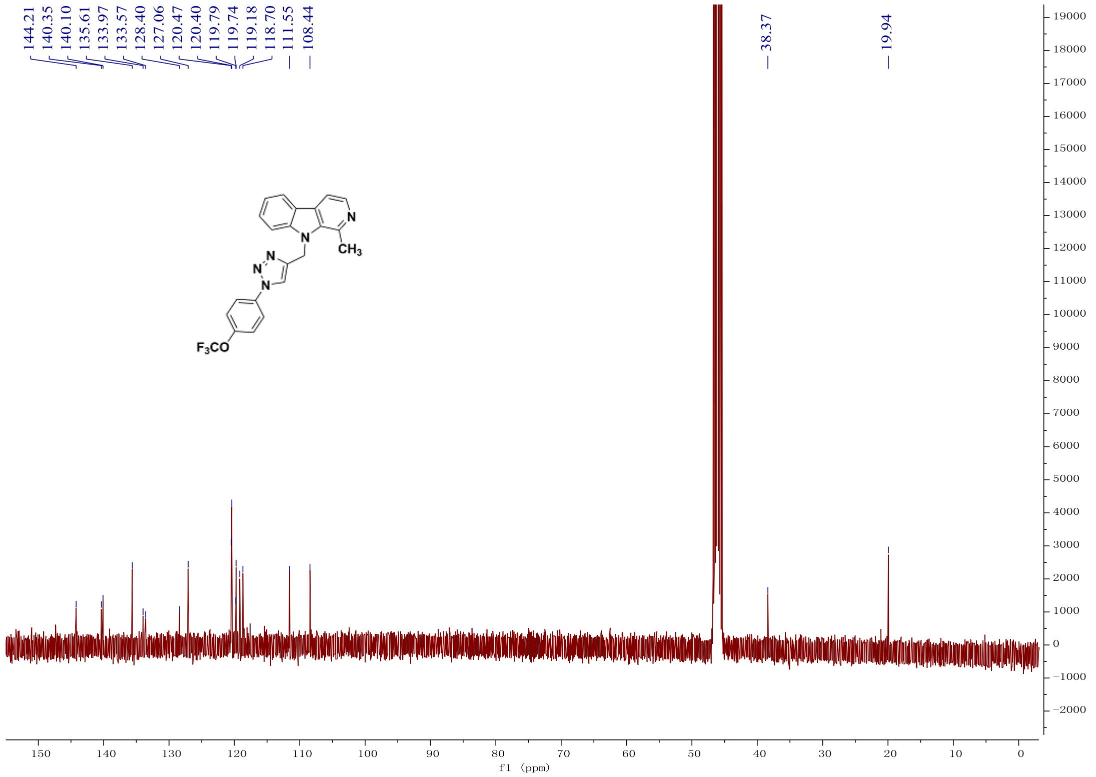


**Figure S8** the $^{1}$H NMR spectrum and $^{13}$C NMR spectrum of compound **4h**

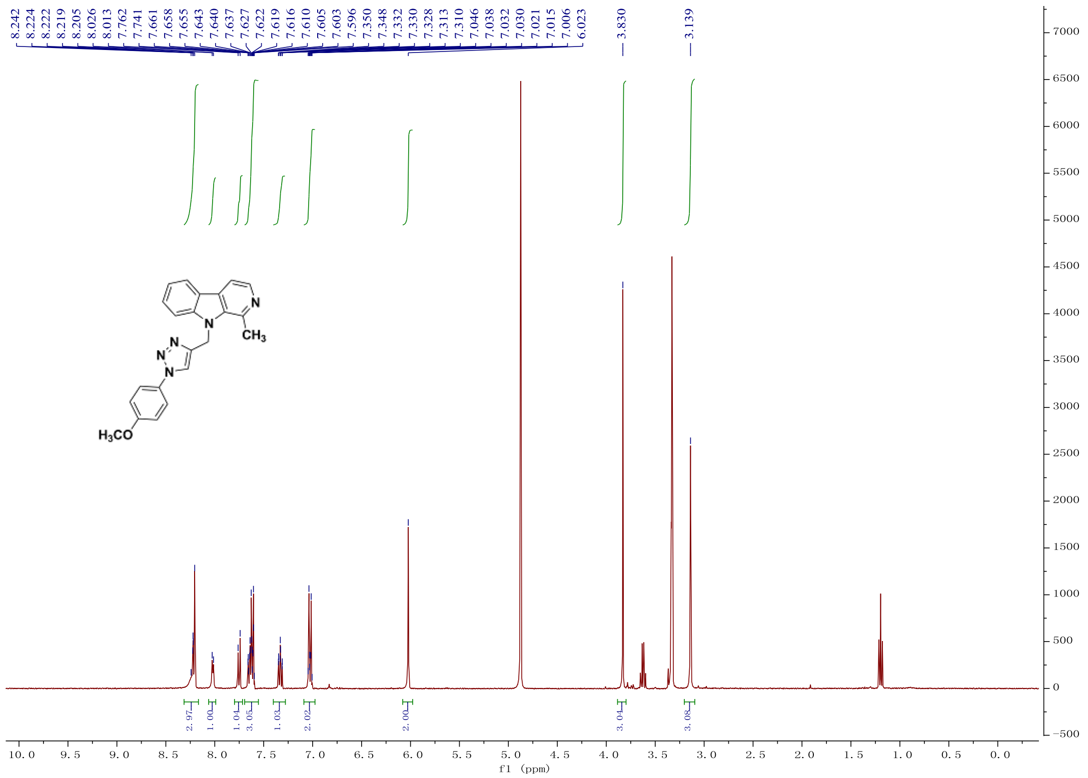


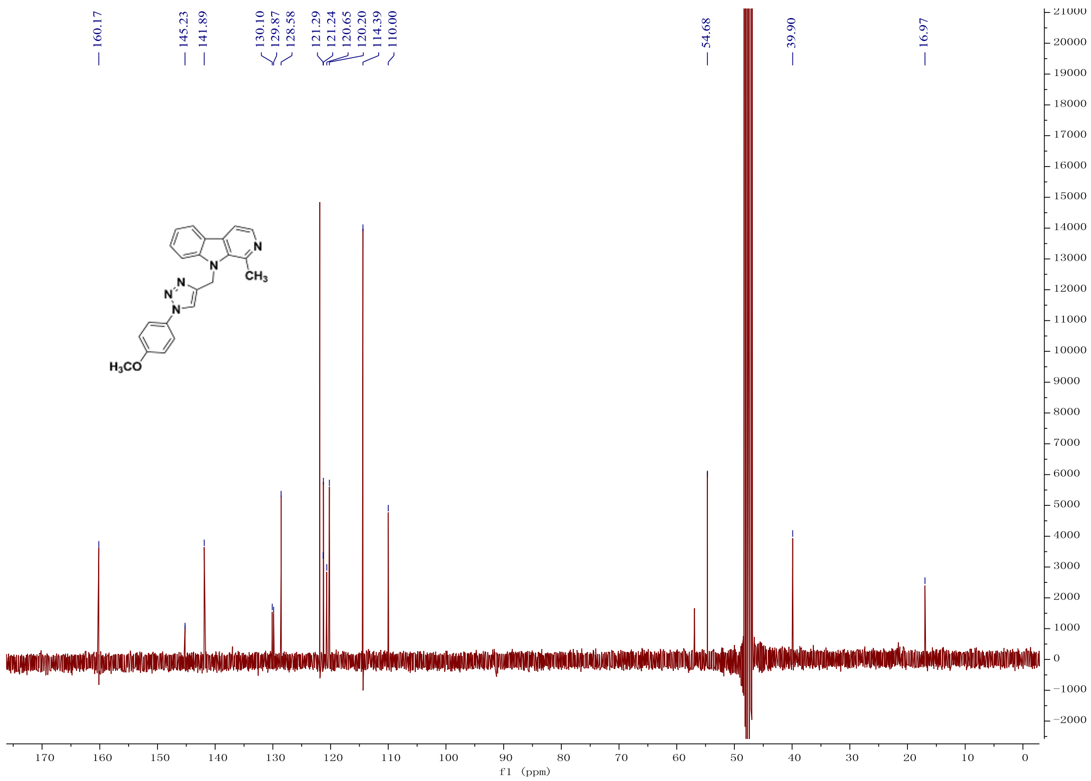


**Figure S9** the $^{1}$H NMR spectrum and $^{13}$C NMR spectrum of compound **4i**

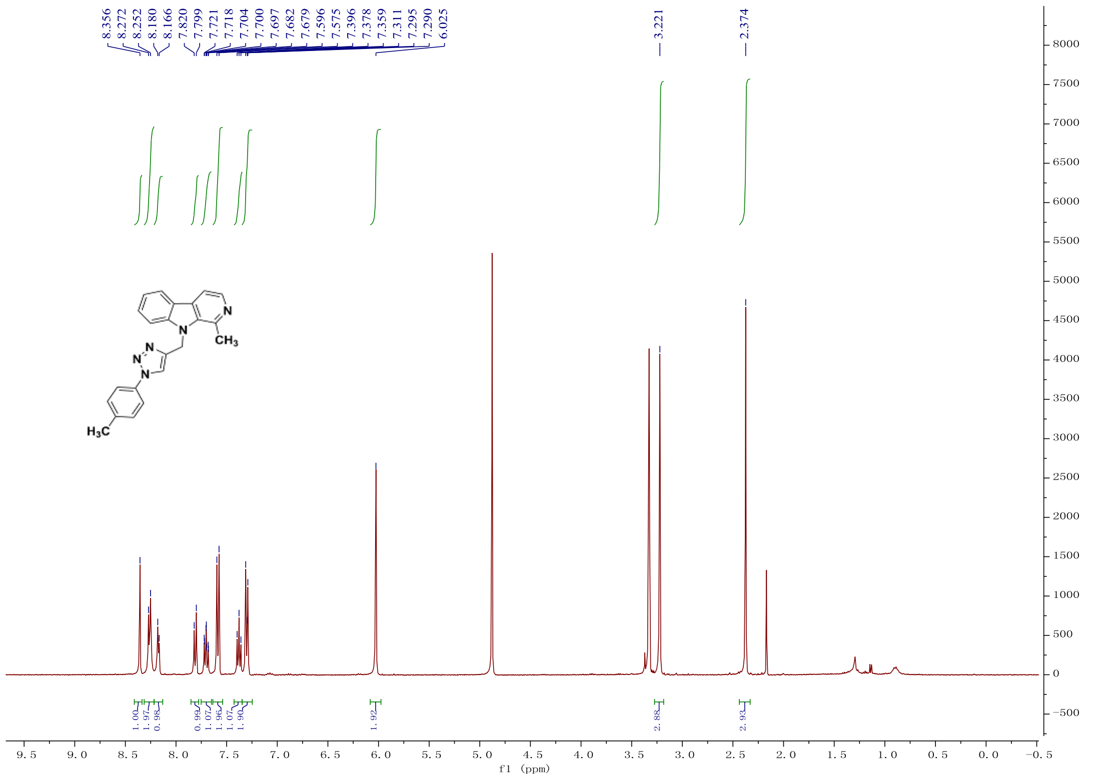

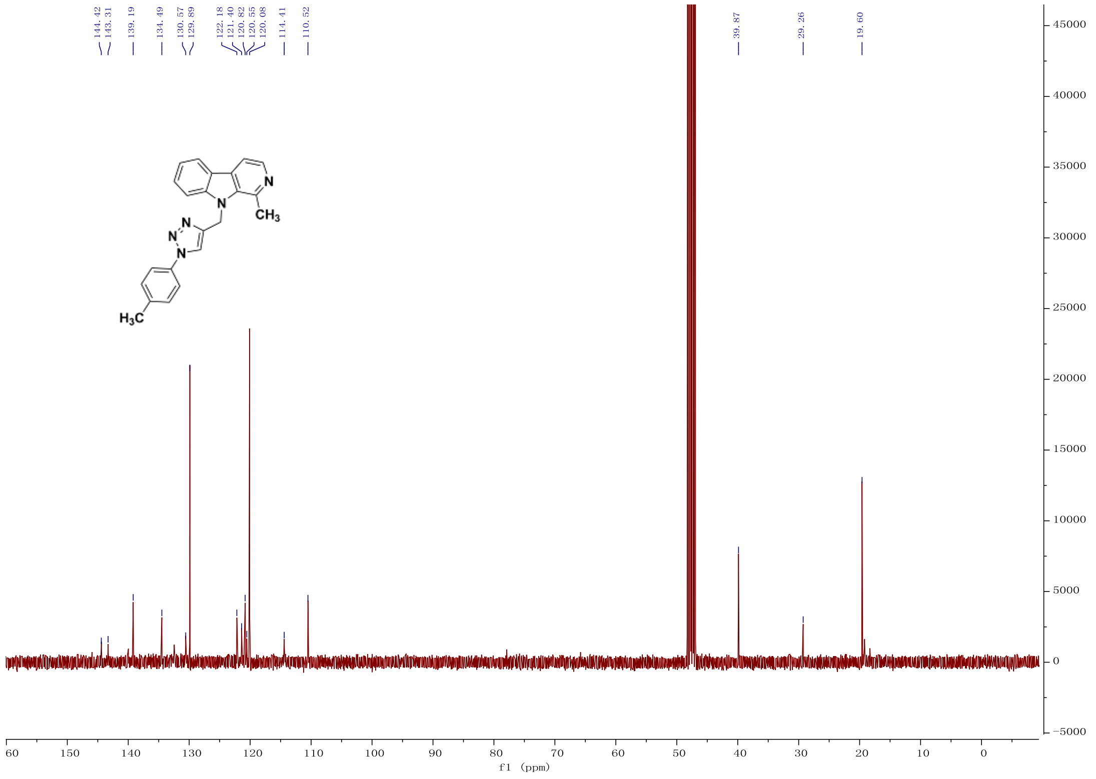

**Figure S10** the $^{1}H$ NMR spectrum and $^{13}C$ NMR spectrum of compound **4j**

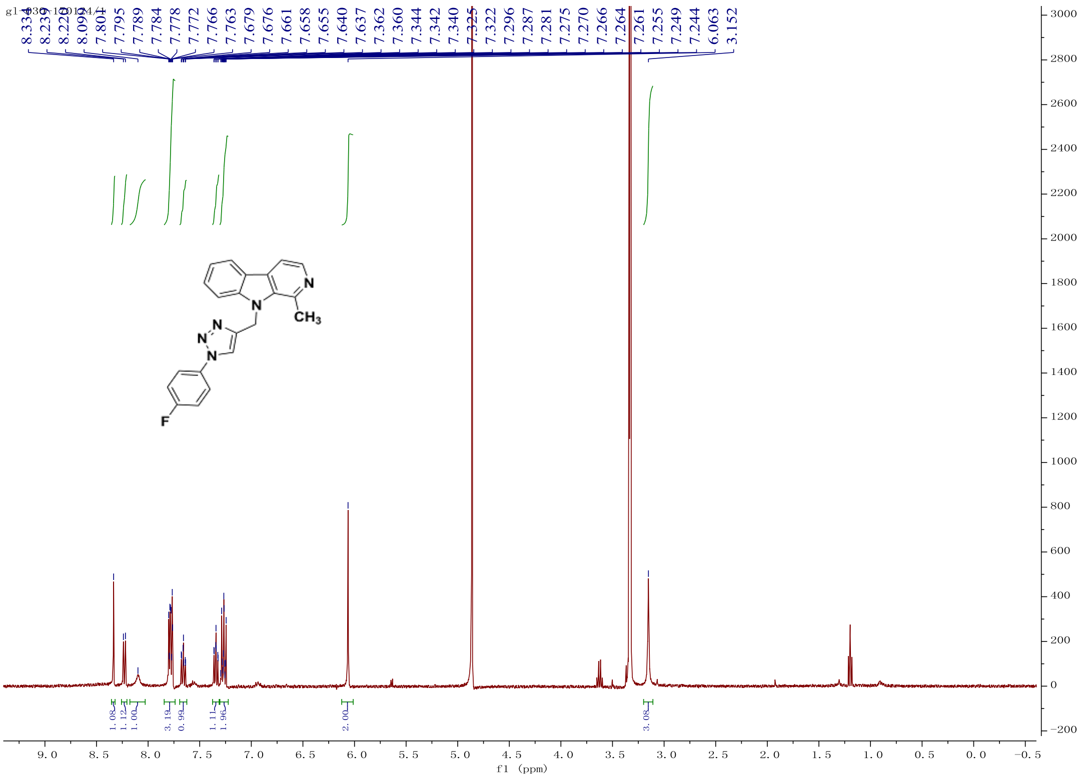


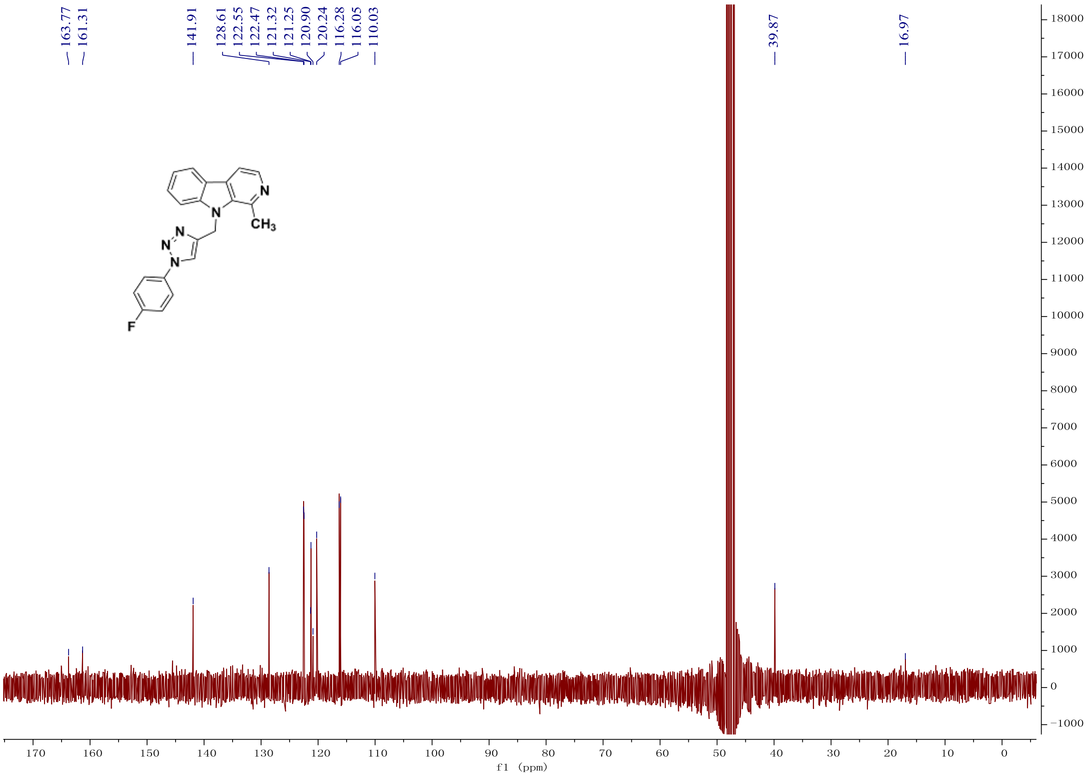


**Figure S10** the $^{1}H$ NMR spectrum and $^{13}C$ NMR spectrum of compound **4k**

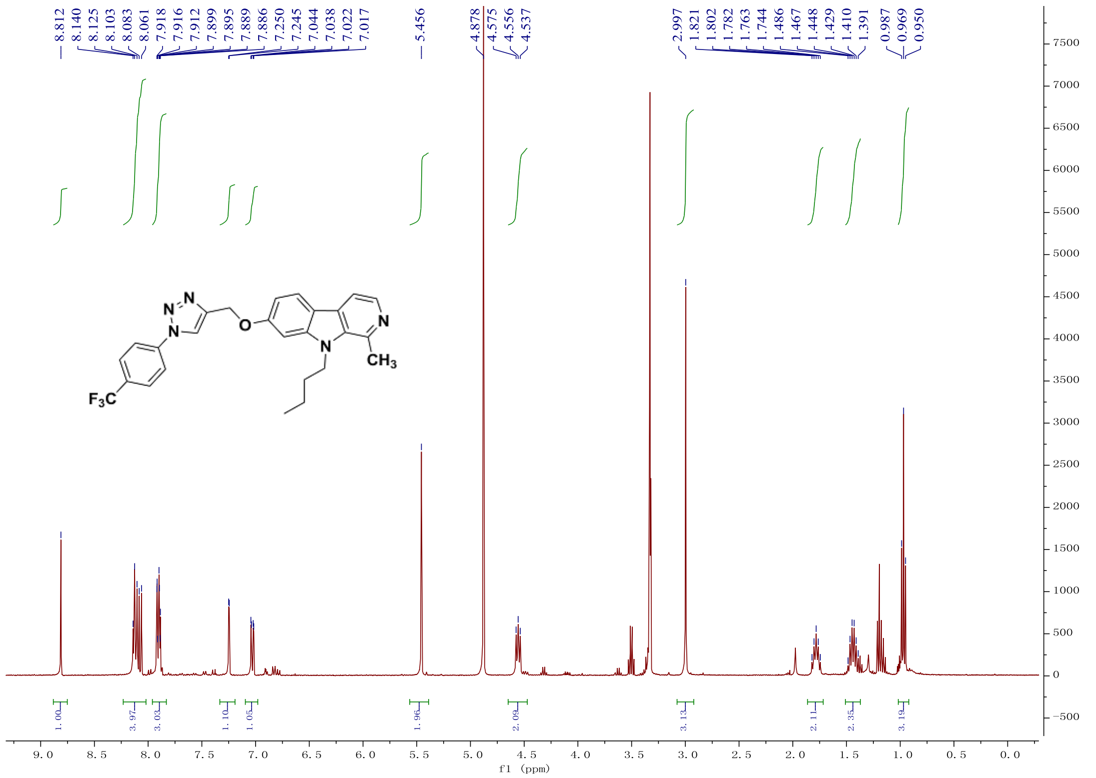


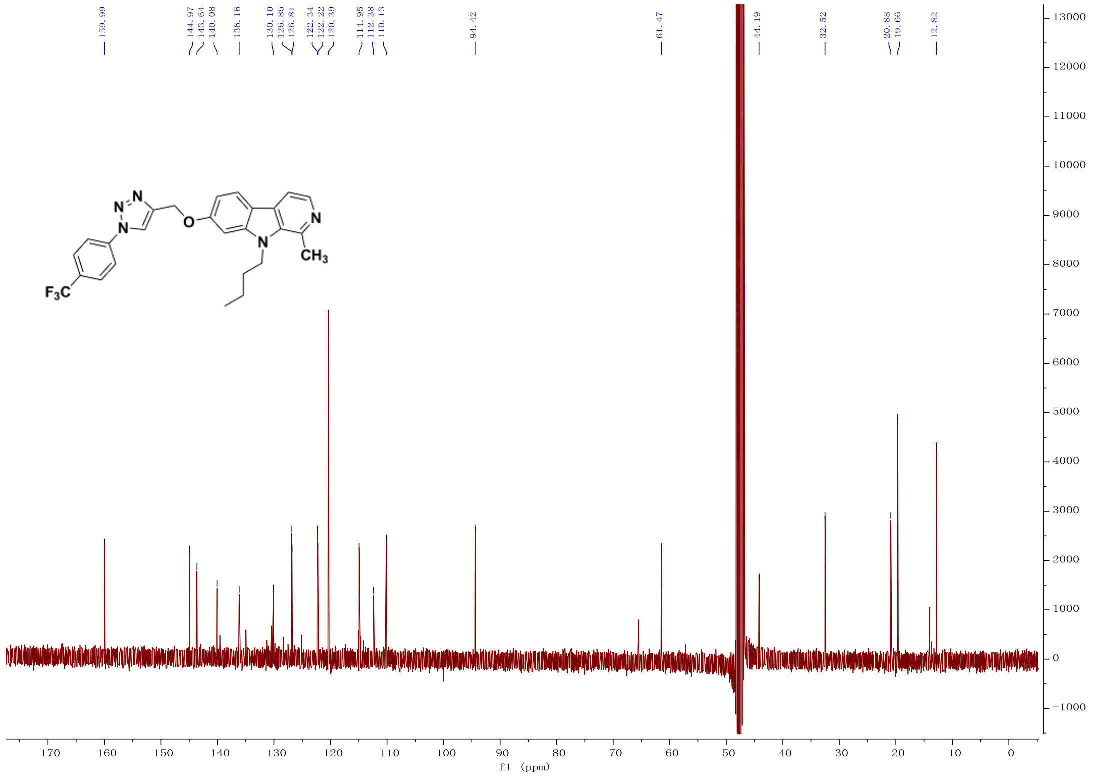


**Figure S12** the $^{1}$H NMR spectrum and $^{13}$C NMR spectrum of compound **8a**

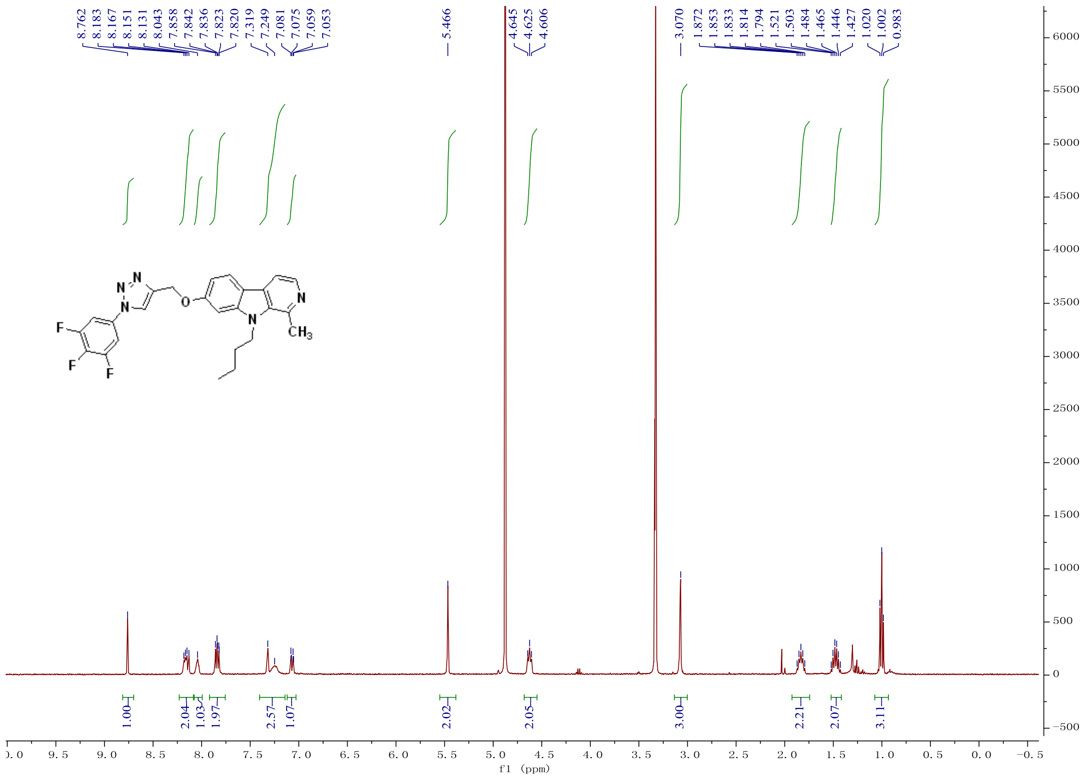


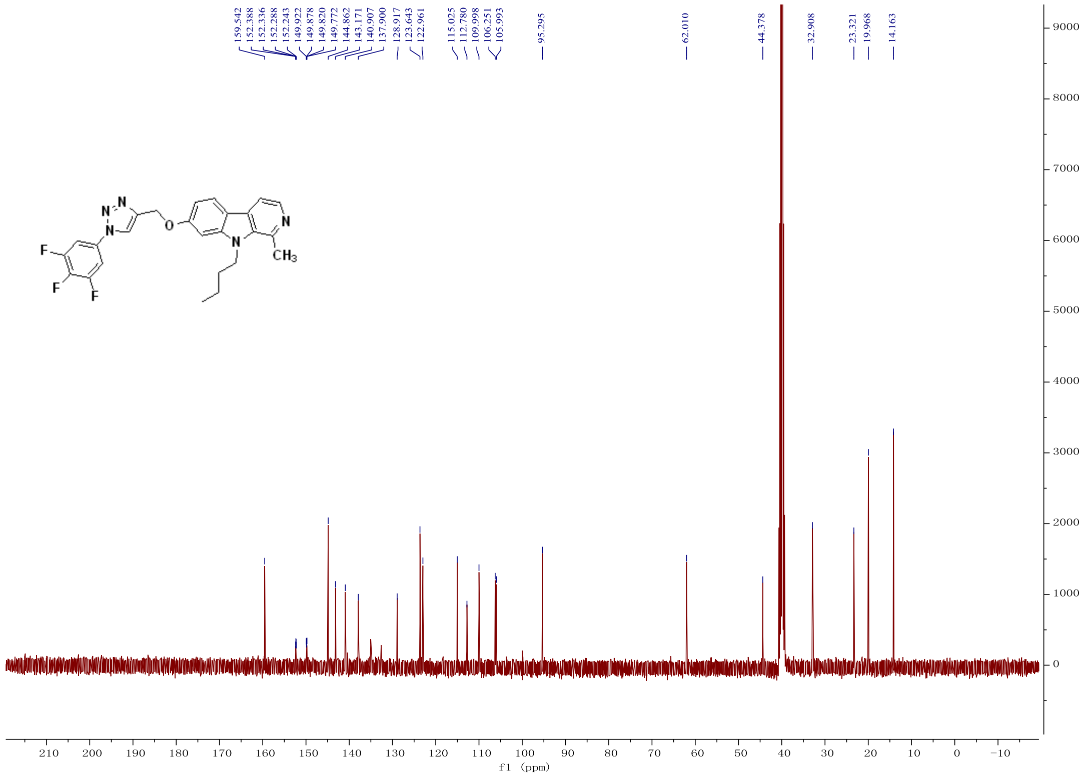


**Figure S13** the $^{1}$H NMR spectrum and $^{13}$C NMR spectrum of compound **8b**

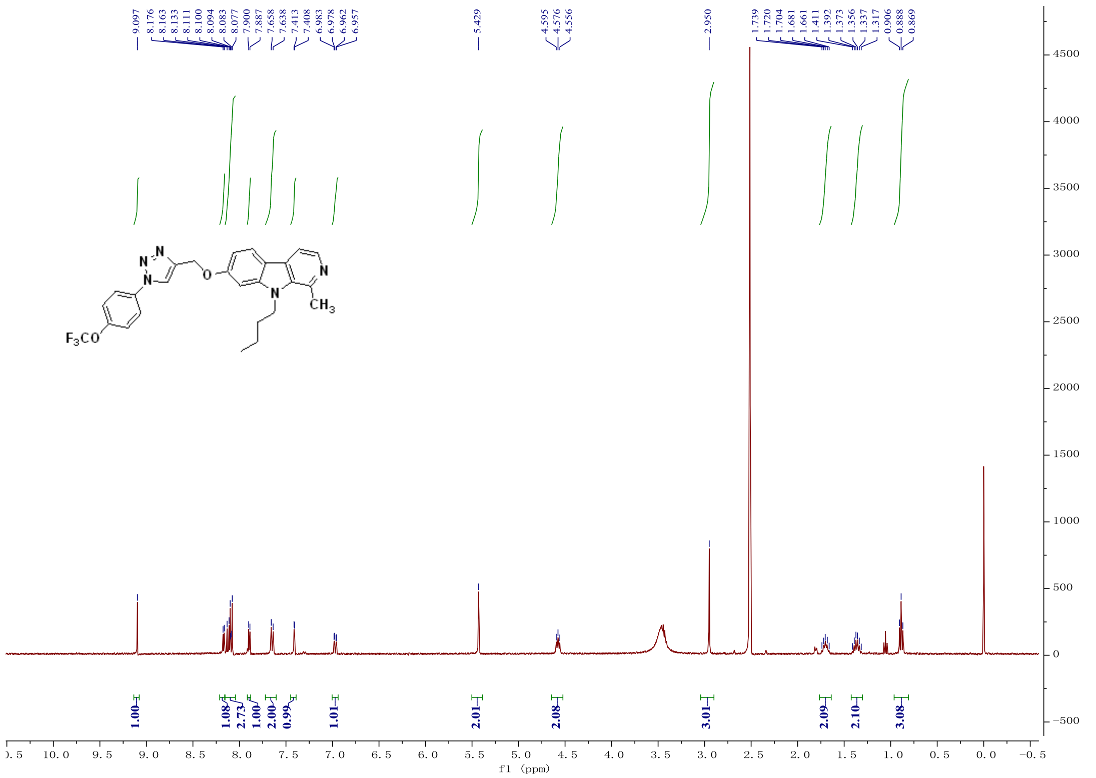


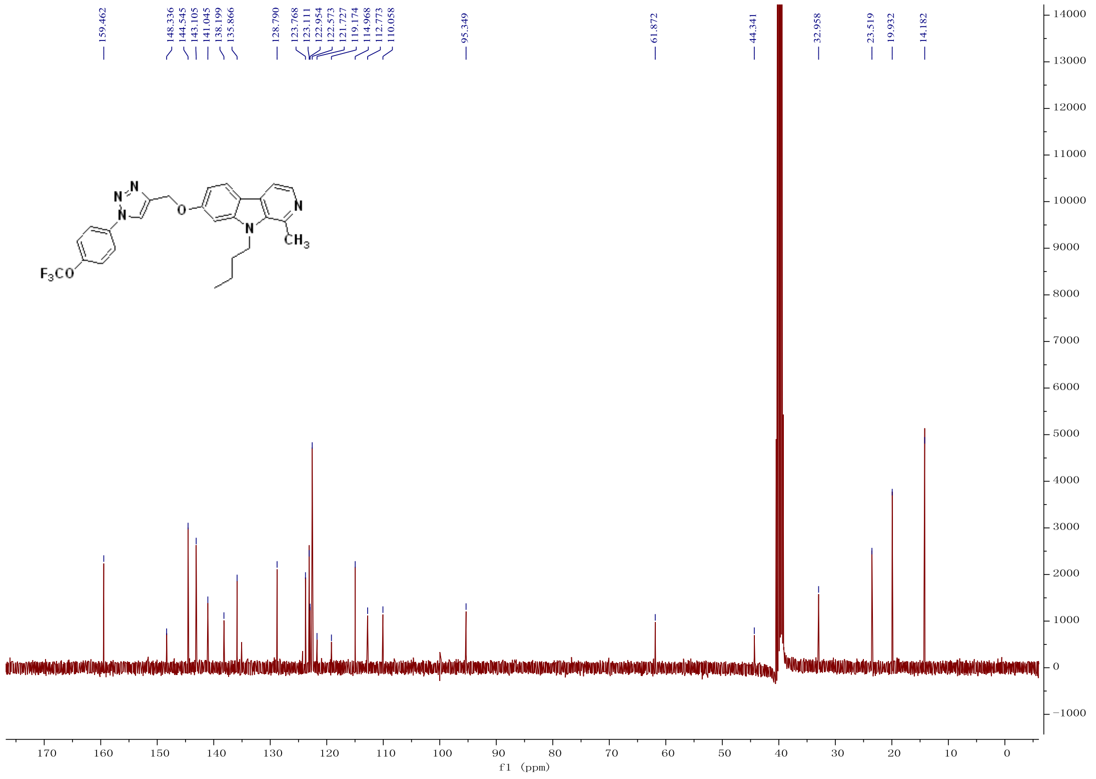


**Figure S14** the $^{1}$H NMR spectrum and $^{13}$C NMR spectrum of compound **8c**

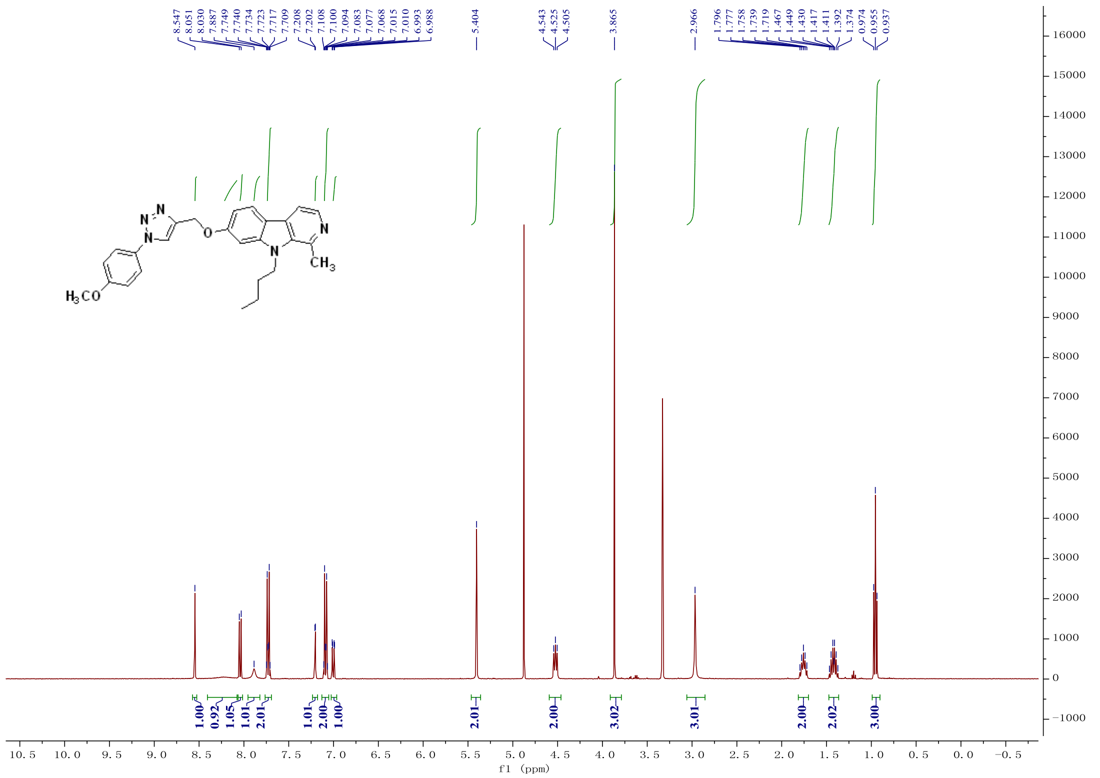

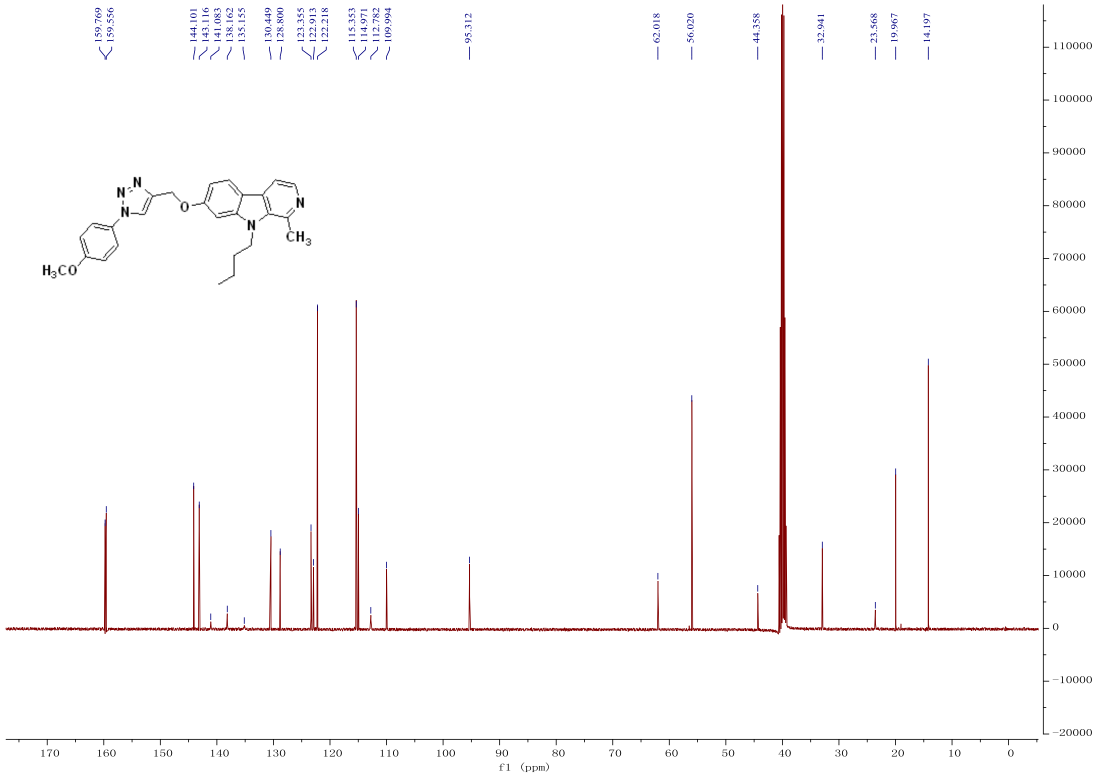

**Figure S15** the $^{1}H$ NMR spectrum and $^{13}C$ NMR spectrum of compound **8d**

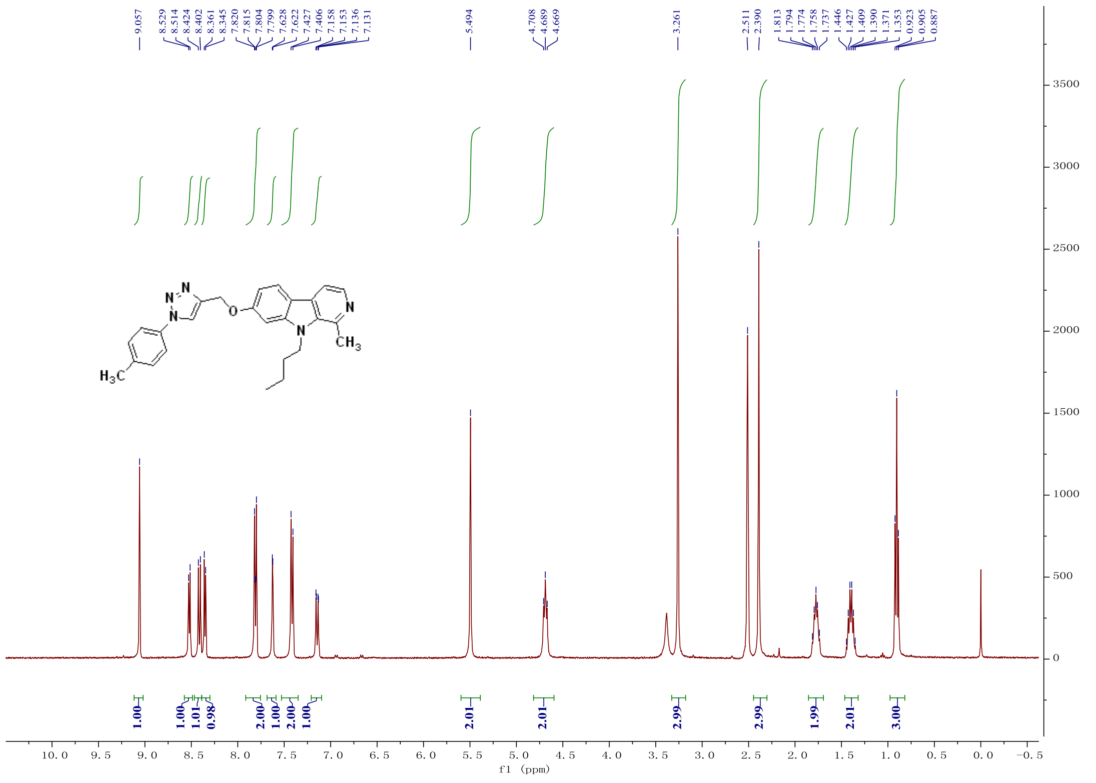

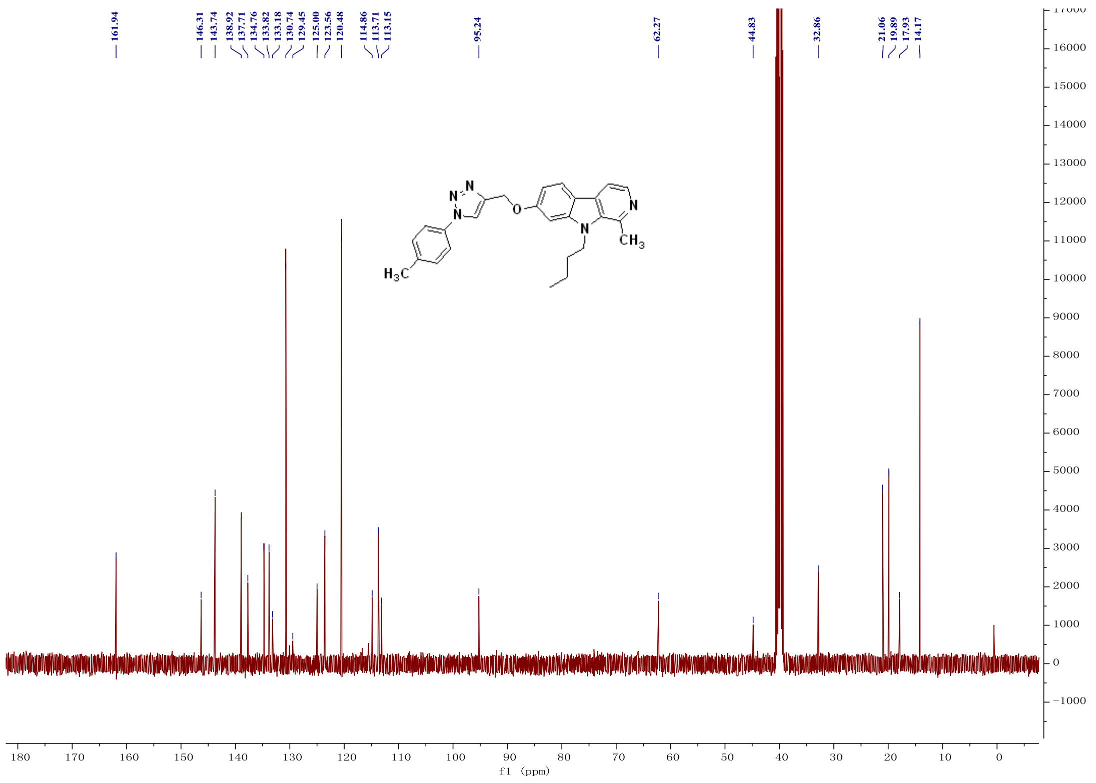

**Figure S16** the $^{1}H$ NMR spectrum and $^{13}C$ NMR spectrum of compound **8e**

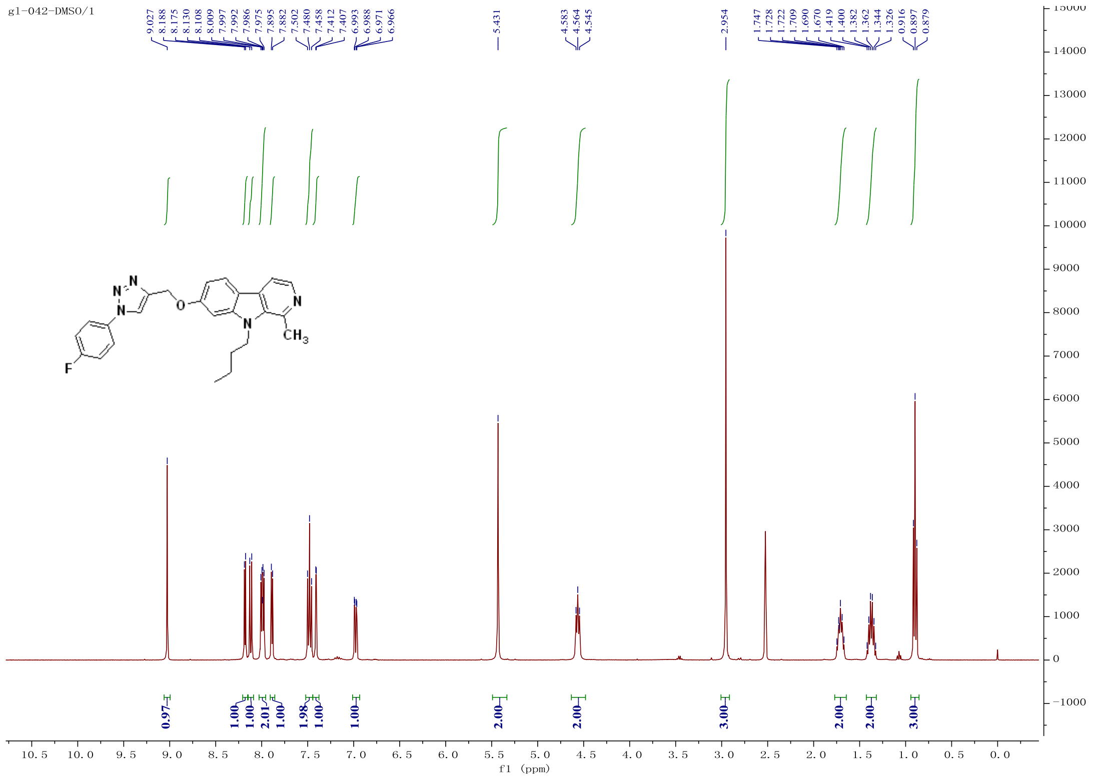


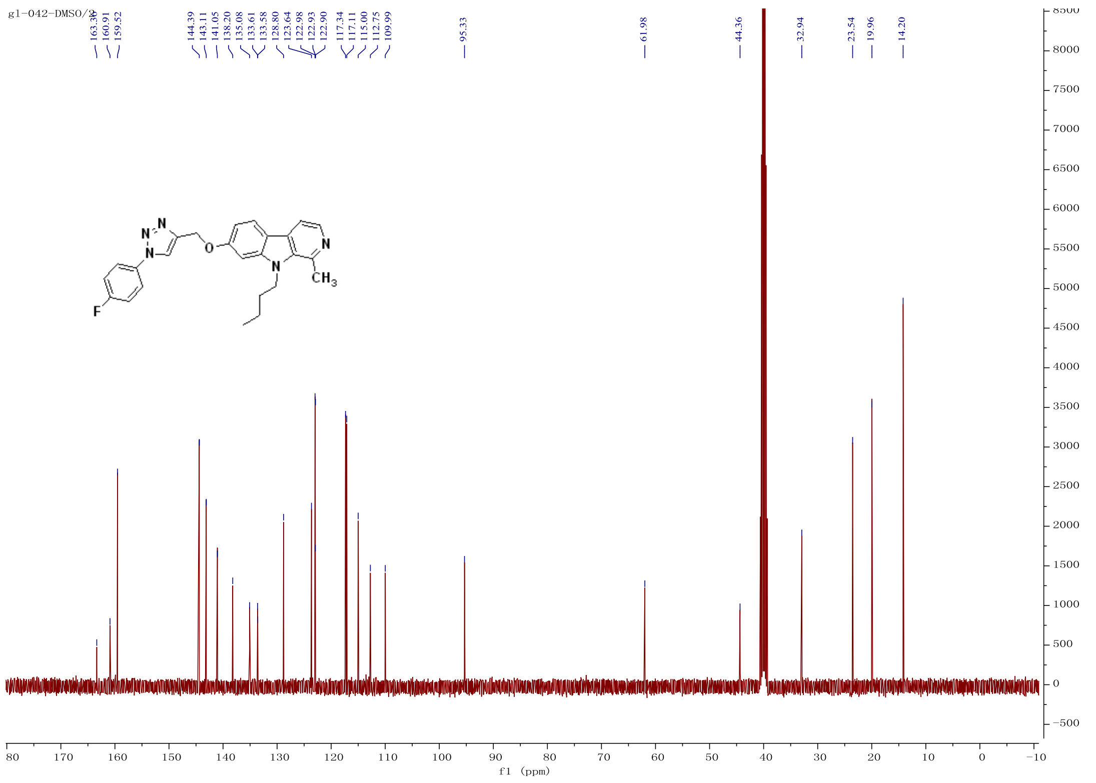


**Figure S17** the $^{1}H$ NMR spectrum and $^{13}C$ NMR spectrum of compound **8f**
